# The triad of maternal gut-breast milk-infant gut microbial transmission in early life as a critical pathway for microbial inheritance

**DOI:** 10.1080/19490976.2025.2574928

**Published:** 2025-11-16

**Authors:** Yanli Du, Jing Cheng, Ruixia Xie, Yongke Zhang, Zhili Huang, Gang Jin, Xiulan Dong, Dayong Sun, Bingxiang Yang, Zongli Han, Xiangyu Wang

**Affiliations:** a School of Medical Technology and Nursing, Shenzhen Polytechnic University, Shenzhen, China; b Department of Obstetrics, The University of Hong Kong-Shenzhen Hospital, Shenzhen, China; c Department of Obstetrics, Peking University Shenzhen Hospital, Shenzhen, China; d Department of Gastroenterology, Shenzhen Second People’s Hospital, The First Affiliated Hospital of Shenzhen University, Shenzhen, China; e School of Nursing, Wuhan University, Wuhan, China; f Department of Neurosurgery, Peking University Shenzhen Hospital, Shenzhen, China; g Clinical Research Center for Digestive Disease, Shenzhen Hospital, Southern Medical University, Shenzhen, China; h Marshall Laboratory of Biomedical Engineering, Shenzhen University, Shenzhen, China

**Keywords:** Maternal-infant microbial transmission, breast milk microbiota, infant gut microbiota, *Bifidobacteria*, breastfeeding patterns

## Abstract

Although maternal microbial inheritance is recognized, the temporal dynamics of how the maternal gut microbiota shapes the infant gut microbiota via specific routes remain unexplored. We performed longitudinal, multi-site microbiota sampling in 30 mother-infant pairs (including 14 exclusive breastfeeding and 16 mixed breastfeeding) from birth to one month, stratified by lactation stages. To trace the origin of breast milk microbiota, we also analyzed colostrum and gut samples from 8 postpartum mothers separated from their infants. Using 16S RNA sequencing, we analyzed microbial diversity and correlations across lactation stages. The results demonstrated that the gut microbiota of exclusively breastfed newborns on the first day primarily originated from breast milk and exhibited remarkably high diversity. We identified maternal gut-breast milk-infant gut transfer as a key pathway for microbial inheritance, with *Bifidobacteria* being a compelling example. These findings provide evidence for this route as a mechanism for inherited microbial diversity.

## Graphical abstract

**Figure:**
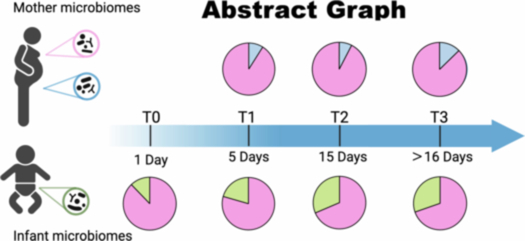

## Introduction

The term “blood connection” is used to describe factors that are passed through the bloodstream between a mother and child. In recent years, scientists have discovered that there is another connection between mother and child: “bacterial inheritance”.^1^^,^^2^

The maternal microbial reservoir is thought to be the source of bacterial lineage transmission.^3^ Microbes transmitted from mothers help in the normal succession of the microbiome and promote the maturation of the neonatal immune system.

In early life, the gut microbiota is a complex and dynamic ecosystem that plays a fundamental role and is an essential driver of a range of immune, metabolic, developmental, and physiological processes that ultimately affecting host health in the long term.^4-6^ The vertical transmission of gut bacteria from mother to their offspring is considered to be a pivotal route for microbiota establishment in newborns, but this transmission is not straightforward. The microbiota that colonizes the adult human intestinal tract is complex, and its structure is specific to each individual. While the intestinal microbiota contains a core community of permanent colonizers, environmentally introduced changes of the microbiota occur throughout adulthood and primarily affect the abundance but not the presence of specific microbial species.^7^ The gut harboring the most heavily populated microbiota in the human body dominates the transmission of mother-to-infant microbiota, ranging from specific bacteria such as *Bifidobacterium* to the overall inherited microbiota.^8^

Studies have also shown that the feeding style is an important factor affecting the establishment of the intestinal flora in infants and that exclusive breastfeeding may be the most important intermediary for the inheritance of mother–infant gut intestinal flora. There has long been a claim that breast milk harbors a core set of bacterial genera (a mean relative abundance of ≥ 1%) dominated by *Pseudomonas*, *Staphylococcus*, and *Streptococcus*, although some of the other members vary across studies, possibly due to the different lactation periods.^9^^,^^10^

Several hypotheses have been formulated to explain the origin of bacteria contained in breast milk.^^11^^ However, predominant theories do not fully explain the microbial diversity of breast milk, particularly the presence of numerous strict anaerobic bacteria. Currently, few studies have addressed the specific relationship between the maternal and neonatal gut microbiota and post-birth development until one month of age. Additionally, no studies have conducted time-series analyses to thoroughly evaluate the mechanisms by which the maternal gut microbiota exerts profound influences on the establishment of the infant gut microbiota.

To thoroughly address the path of bacterial succession, we longitudinally sampled the microbiomes of 14 exclusive breastfeeding (EB) and 16 mixed breastfeeding (MB; more than 70% breast milk supplemented with less than 30% formula milk) mother‒infant pairs across multiple time points and various body sites sampled from birth to one month postpartum. In addition, to trace the source of the maternal breast milk microbiota, we selected 8 mothers who were immediately separated from their newborns after delivery and collected both colostrum and intestinal samples for a rigorous one-to-one source tracing analysis. Microbial identification technology based on ribosomal RNA gene pyrosequencing was used to continuously and systematically analyze the diversity and correlation of maternal intestinal, breast milk and infant intestinal flora at different time points. This approach provides a solid foundation for identifying the maternal gut-breast milk-infant gut microbiota triad as a significant pathway for microbial inheritance. The findings reveal the patterns of co-occurrence of gut and milk bacteria and underscore the significant impact of feeding methods on the infant gut microbiome.

## Materials and methods

### Participants

**Figure 1.**
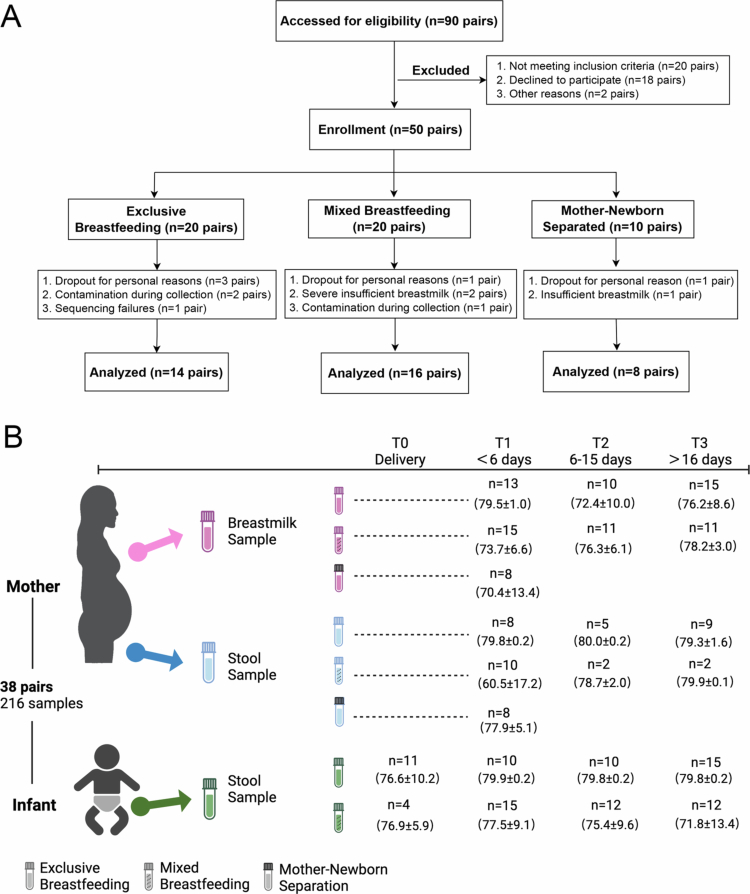
Longitudinal sampling and 16sRNA sequencing of the microbiome of mother-infant pairs. Samples were collected from 30 mother-infant pairs in the MNT group and 8 mothers from the MNS group after delivery from the mother’s stool (blue), the mother’s breast milk (pink) and the infant’s stool (green) according to the lactating stage. All the samples were 16S RNA sequenced, and the average depth (in kb) of the quality-controlled and human DNA-free samples was reported. T0: within 24 hr; T1: ≤ 5 d; T2: 6–15 d; T3: 16–30 d.

This study was conducted from January to December 2022 at the University of Hong Kong-Shenzhen Hospital in Shenzhen, China. The participants were divided into groups based on their breastfeeding and cohabitation patterns. The flowchart for participant recruitment is presented in Figure 1. The mother‒newborn together group (MNT) included mother-infant pairs who were exclusively breastfeeding (EB) or mixed breastfeeding (MB). For the MNT group, 30 healthy volunteers (14 EB and 16 MB) who met the inclusion and exclusion criteria were analyzed in the study. The inclusion criteria were as follows: ≥20 y old, healthy during pregnancy, without any common pregnancy complications, did not use antibiotics during pregnancy, full-term delivery, settled in Shenzhen, Han nationality, and normal lactating woman. The exclusion criteria were: pregnant women with gestational diabetes, pregnancy-induced hypertension syndrome, acute communicable diseases, and ethnic minorities who were separated from their newborns. The mother‒newborn separation group (MNS) had the same inclusion criteria but with newborns who were immediately transferred to the neonatal intensive care unit (NICU) after birth for monitoring or treatment due to small-gestational-age or extended vaginal delivery, resulting in the separation of mother and baby. Thus, 8 volunteers’ colostrum samples were collected from mothers who were unable to nurse their newborn babies.

The participants completed a questionnaire that included basic maternal information (name, age, telephone number and address), pregnancy and childbirth information (number of pregnancies, parity, the number of gestational weeks at delivery, education level, diet, delivery method, and prophylactic use of antibiotics during the delivery), as well as information about the infant, including gender, height, and weight (Table 1 and Supplementary Table S1).

**Table 1.** Average demographics of the participants.

Variable	Data		
	MNT		MNS
	EB (*n* = 14)	MB (*n* = 16)	MNS (*n* = 8)
Mother’s age (years), Mean (SD)	32.0 (4.0)	32 (3.6)	32 (3.5)
Mother’s weight gain during pregnancy (kg), Mean (SD)	10.7 (3.5)	13.0 (3.2)	10.5 (2.7)
Pregnancy number, X (*N*)	1 (3) 2 (8) 3 (3)	1 (6) 2 (7) 3 (3)	1 (7) 2 (1) 3 (0)
Delivery mode (*N*)	Vaginal (9) C-section (5)	Vaginal (12) C-section (4)	Vaginal (6) C-section (2)
Intrapartum antibiotics during delivery (*N*)	Yes (6) No (8)	Yes (6) No (10)	Yes (3) No (5)
Infant gender (*N*)	Male (9) Female (5)	Male (10) Female (6)	Male (3) Female (5)
Newborn weight (kg), Mean (SD)	3.5 (0.3)	3.2 (0.4)	2.8 (0.5)

Note: MNT: mother newborn together; MB: mixed breastfeeding; EB: exclusive breastfeeding; MNS: mother-newborn separated.

### Sample collection

For the EB and MB groups, breast milk was collected according to the lactating stages after delivery, which included the colostrum stage (T1, 0–5 d), transitional-milk stage (T2, 6–15 d) and mature-milk stage (T3, 16–30 d). The mothers’ feces were collected at each stage or only at the colostrum stage (T1). The babies’ feces were collected at four time points: after birth (T0, 0–24 hr/meconium) and at the above three lactating stages (T1, T2 and T3). For the MNS group, colostrum and mothers’ feces were collected from all 8 lactating mothers at T1 during their hospitalization.

Breast milk was collected by an International Board-Certified Lactation Consultant using an aseptic protocol. Briefly, 8–10 ml of sterile water was used to wash one nipple and the surrounding nipple skin (NS). Then, the first drop of breast milk was discarded with an aseptic yarn block, and the breast milk (3–5 ml) was manually collected into an enzyme-free, aseptic centrifugal tube while wearing sterile gloves. Feces were collected using sterile dry stool collection tubes. After sealing the tube with sealing film, the breast milk and feces samples were quickly frozen in liquid nitrogen and then transferred to a -80 °C freezer for storage. Total genomic DNA was extracted shortly after collection to prevent the microbiota from replicating.

### Ethics statement

This study was conducted according to the guidelines set forth in the Declaration of Helsinki. The experiments in this study were approved by the Ethics Committee of the University of Hong Kong-Shenzhen Hospital (No. [2021]214). Written consent was obtained from each volunteer.

### Sample processing and 16S RNA sequencing

Bacterial DNA was extracted from the breast milk and fecal samples using the TGuide S96 Magnetic Soil/Stool DNA Kit (Tiangen Biotech (Beijing) Co., Ltd. China). PCR amplification was conducted with barcoded specific bacterial primers targeting the variable region 3–4 (V3–V4) of the 16S rRNA gene. The primers used were F: 5'-ACTCCTACGGGAGGCAGCA-3' and R: 5'-GGACTACHVGGGTWTCTAAT-3'. The PCR reaction (total reaction volume of 10 μl) included: DNA template 2.5–4 ng, *Vn F (10 μM) 0.3  μl, *Vn R (10  μM) 0.3 μl, KOD FX Neo Buffer 5 μl, dNTP (2 mM each) 2 μl, KOD FX Neo 0.2 μl, and ddH2O up to 10 μl. The amplification conditions were as follows: an initial denaturation at 95 °C for 5 min, followed by 25 cycles of 95 °C for 30 s, 50 °C for 30 s, and 72 °C for 40 s; and a final extension at 72 °C for 7 min. The PCR-amplified products were mixed and purified using an Omega DNA purification column (Norcross, Georgia, USA). The mixed PCR-amplified products were then purified and recovered using 1.8% agarose gel electrophoresis. Sequencing libraries were constructed, and paired-end sequencing was performed on an Illumina NovaSeq6000 platform at Biomarker Technologies Co., Ltd. (Beijing, China) according to standard protocols. Paired-end reads were merged using FLASH v1.2.7,^12^ and tags with more than six mismatches were discarded. The merged tags with an average quality score < 20 in a 50-bp sliding window were determined using Trimmomatic,^13^ and those shorter than 350 bps were removed. After quality control, the data were denoised, and amplicon sequence variants (ASVs) had a clustering similarity of 100% using QIIME2^14^ DADA2 (version 2020.6).^15^ The raw reads were processed with Trimmomatic v0.33 to filter out low-quality fragments, followed by adapter and primer removal using Cutadapt 1.9.1 to obtain clean reads. The sequences were aligned to the human genome using Bowtie2. Taxonomy was assigned to all ASVs by searching against the Silva databases (Release 138) using QIIME2 software.

### Data analysis

Statistical analyses were performed in R (Version 4.0.1). Microbiota profiles were included in the estimation of alpha diversity (referring to diversity within a particular region or ecosystem) and beta diversity (comparing the similarity of species diversity among different samples) as described by Liu et al.^16^ The alpha diversity metrics, including the Shannon, Simpson, Chao 1 and Richness indices were first assessed for differences among the MNS, EB, and MB groups using the Kruskal–Wallis test. Pairwise comparisons were then performed using the Wilcoxon rank-sum test. Dissimilarities were estimated with the Bray–Curtis dissimilarity index and UniFrac indices^17^ and analyzed with unconstrained principal coordinate analysis. Permutational multivariate analysis of variance was used to describe the strength and significance of categorical factors in determining the variation of ecological distances.

Differential abundance analysis between groups was performed using the edgeR package in R. The statistical model implemented in edgeR was applied to test for significant differences in species abundance, with a significance threshold set at *P* < 0.05. Spearman's correlation was used to calculate the correlation at the genus level between the colostrum of each mother and her intestinal microbiota in the mother–infant separation group. The correlation between *Bifidobacterium* abundance and time in the EB and MB groups was also assessed using Spearman's method.

Furthermore, Spearman's correlation was employed to analyze the relationship between *Bifidobacterium* in maternal colostrum and infant meconium.

The impact diagram was generated using the R package ggalluvial (v0.12.5), illustrating the relative abundance changes in the top 20 genera with the highest genus-level abundance, as well as the remaining genera (referred to as “Other” for all genera excluding the top 20) across different groups and time points. The fast expectation-maximization microbial source tracking (FEAST) method^18^ was used to assess the contribution of each source to the target microbiome. The R package FEAST (v.0.1.0) was used to compute the contribution of each maternal fecal sample to its corresponding breast milk microbiome, as well as the contribution of each breast milk sample to the infant fecal microbiome. The OTU table and sample metadata were utilized as input, with EM_iterations set to 1000 for the purpose of achieving algorithm convergence. To assess the stability of the estimated transmission proportions, 100 bootstrap resamplings were performed, from which 95% confidence intervals were calculated. Adobe Illustrator was used to visualize the results.

## Results

### Sample collection and quality control

To validate the gut‒breast axis hypothesis, we collected and analyzed samples from postpartum mothers and their newborns. The mother–infant separation (MNS) group included 8 mothers aged 26–36 y who were separated from their newborns immediately after delivery due to neonatal or maternal complications, while the MNT (mother–infant together) group included 30 mothers aged 23–39 y and their newborns. The MNT group was further divided into an exclusive breastfeeding (EB) group and a mixed feeding (MB) group. The MB group primarily breastfed (≥70%) but also supplemented with formula milk. Samples from the MNS group were taken only from the mothers at the colostrum stage (T1; 0–5 d), while samples from the EB and MB groups were taken from the mothers and/or newborns at the meconium (T0), colostrum (T1), transitional milk (T2; 6–15 d), and mature milk (T3; 16–30 d) stages (Figure 1). Additional demographic details are presented in Table 1 and Supplementary Table S1. The extracted DNA concentration, raw sequencing reads, clean reads (after quality control) and the alignment ratio to the human genome are provided in Supplementary Table S2. These results revealed an extremely low proportion of human-derived sequences across samples (12 samples < 0.2%, remaining 204 samples = 0%). The rarefaction curves (Supplementary Figure S1A) and species accumulation curves (Figure S1B) further validated the reasonableness of our sequencing depth and sample size.

### Comparative analysis of the alpha and beta diversity of maternal gut, breastmilk and infant gut microbiota

To evaluate the bacterial species in maternal and infant samples, we performed 16S rRNA sequencing. A total of 74951, 75530 and 77231 sequencing reads were generated and clustered into 7565, 7998 and 7778 OTUs, respectively, in maternal gut, breastmilk and infant gut groups. Among them, 91.0%, 90.7% and 90.9% of the OTUs were classified at the genus level.

**Figure 2.**
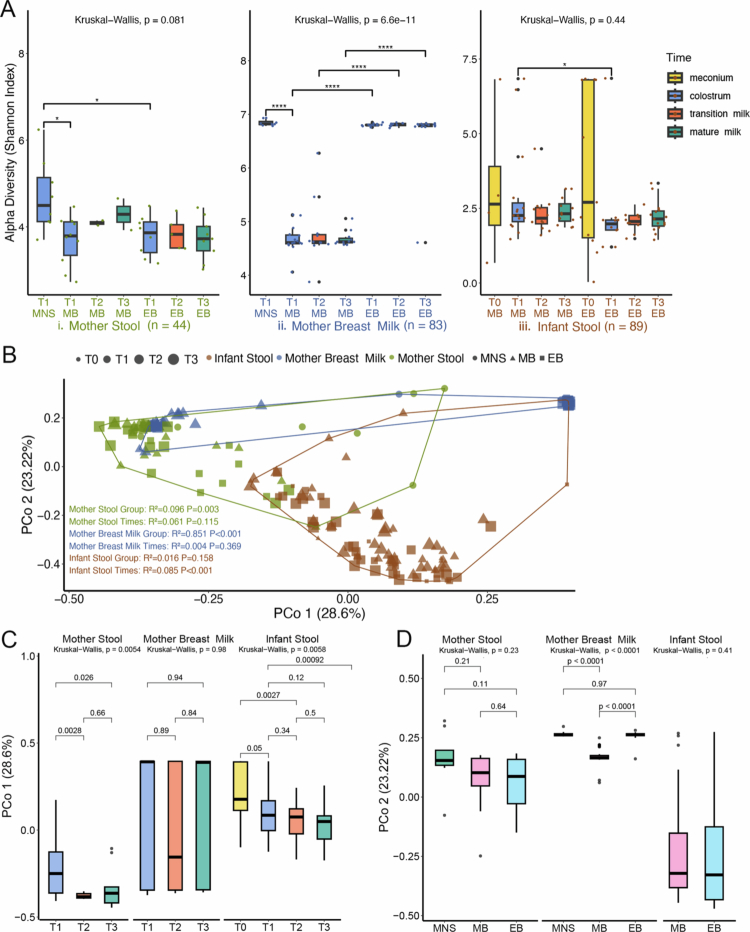
Maternal and infant microbial diversity and composition of the dominant bacteria. (A) Alpha diversity distributions for each sample type and time point according to Shannon diversity scoring. Differences within each sample source were first tested by the Kruskal‒Wallis test, followed by pairwise Wilcoxon tests to assess differences between groups(*p < 0.05, ***p < 0.001). (B)–(D) Comparison of beta diversity in microbiota from different maternal and infant sites across multiple time points. (B) Unconstrained principal coordinate analysis (PCoA) based on the UniFrac distance. The text annotations show PERMANOVA results for the effects of sample source (“Group”) and time (“Time”) on differences across sample sources. (C) PCo1 was used to compare the impact of lactation stages on the microbiota. (D) PCo2 was used to compare the impact of feeding patterns on the microbiota. The percentage of variation indicated on each axis of (C) and (D) corresponds to the fraction of the total variance explained by the projection. Statistical significance among multiple groups was assessed using the Kruskal‒Wallis test, followed by pairwise comparisons with the Wilcoxon rank‒sum test.

To compare the phylogenetic profiles between the samples, we conducted comparative *α* diversity analysis using the Shannon, Simpson, Chao 1 and Richness indices separately (Table 2). As shown in Figure 2A (mother stool comparisons) and supplementary Figure S2, the *α* diversity for the maternal gut samples was significantly greater in the MNS group at T1 than in the MB or EB group at T1 (Shannon, Chao 1 and Richness indices). There were no significant differences in the *α* diversity scores at the T1–T3 lactation stages within the EB and MB groups. For the breast milk samples (Figures 2B and S2), the MNS group presented a greater diversity score than did the MB group (*P* ≤ 0.001) but a similar score to that of the EB group in the T1 stage. The microbial types and richness in the EB group were significantly greater than those in the MB group, suggesting that the feeding method had a significant impact on the microbiota diversity in the breast milk, which is likely related to the duration and frequency of infant sucking at the mother's breast in the EB group. However, there were no differences in the *α*-diversity of the microbiota across the three stages within the EB and MB groups, suggesting that the types and abundance of the microbiota do not change over time. For the infant stool samples (Figures 2C and S2), no significant changes in *α*-diversity were observed from the meconium stage to the mature milk stage (T0–T3) in either the EB or the MB group (Shannon, Simpson and Richness indices). This finding suggests that the gut microbiota in these infants is likely to be established at an early stage. Compared with the EB and MB groups at the same lactation stage, the Shannon and Simpson indices showed almost no significant difference, while Chao1 and Richness showed that the microbiota composition of the MB group was significantly greater than that of the EB group at every stage.

Next, we performed principal coordinate analysis of the β diversity. To distinguish the composition of the microbiota of MNS, EB and MB, PERMANOVA was performed. Results showed that there were significant differences in maternal stool (*p* = 0.003) and maternal breast milk (*p* < 0.001) among the three groups (Figure 2B). There was significance in infant stool between EB and MB affected by lactating stages (*p* < 0.001). For a more detailed understanding of potential differences in β-diversity over time, we used PCo1 to compare the impact of lactation stages on the microbiota. A significant difference in the diversity between T1 and T2/T3 was observed for the maternal gut microbiota (Kruskal-Wallis test *P*-value = 0.05). Furthermore, the diversity of infant meconium microbiota (T0) also showed significant differences compared to T2 and T3 (Kruskal-Wallis test *p*-value < 0.05), though no effects of the lactation stages were observed on the diversity of the breast milk microbiota (Kruskal-Wallis test *p*-value > 0.05) (Figure 2C). These results suggest that the maternal and infant gut microbiota, but not the breast milk microbiota, change according to the lactation stage.

To further understand the impact of feeding patterns on the microbiota, we used PCo2 to compare the diversity between samples. The β-diversity of breast milk in the MNS group was significantly greater than that in the MB group (Kruskal‒Wallis test *P*-value ≤ 0.001), though no significant differences were detected between the MNS and EB groups (Kruskal‒Wallis test *P*-value = 0.97) (Figure 2D). These results suggest that the diversity is significantly influenced by the feeding patterns for the breast milk microbiota but not the maternal or infant gut microbiota. The differences in sample types along the PCoA1 axis (Figure S3A) and the differences in lactation stages along the PCoA2 axis plot (Figure S3B) were also analyzed.

**Table 2.** Comparative analysis of alpha-diversity indices: significant differences across sample types and time points.

Sample source	Subgroup comparisons	Shannon index	Simpson index	Chao 1 index	Richness index	Significant indices (*n*)
Mother stool	T1 MNS vs T1 MB	*	*	***	**	4
	T1 MNS vs T1 EB	*	NotSig	***	***	3
	T1 MB vs T1 EB	NotSig	NotSig	**	**	2
Mother breast milk	T1 MNS vs T1 MB	****	****	****	****	4
	T1 MB vs T1 EB	****	****	****	****	4
	T2 MB vs T2 EB	****	****	****	***	4
	T3 MB vs T3 EB	****	****	****	****	4
Infant stool	T1 MB vs T1 EB	*	NotSig	**	**	3
	T2 MB vs T2 EB	NotSig	NotSig	**	**	2
	T3 MB vs T3 EB	NotSig	NotSig	*	**	2
	T0 MB vs T2 EB	NotSig	NotSig	*	NotSig	1

Note: MB: mixed breastfeeding; EB: exclusive breastfeeding; MNS: mother‒newborn separated; NotSig: no significance; *p < 0.05, **p < 0.01, ***p < 0.001, ****p < 0.0001.

### Differences in the relative abundances of predominant microbiota taxa

**Figure 3.**
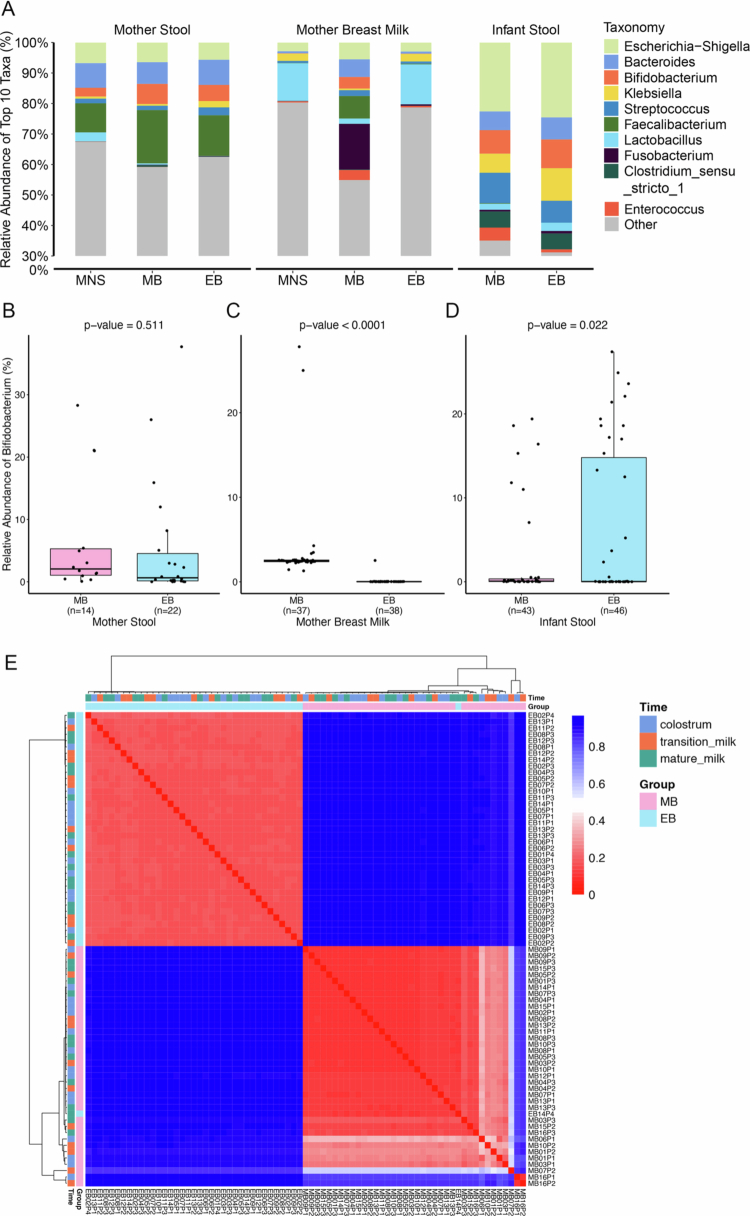
Comparative analysis of the effects of different feeding methods on the composition of *Bifidobacterium* and other microbiota in the maternal gut, infant gut and breast milk. (A) Species composition plot. The top 10 most abundant bacterial genera in samples from different body sites are shown. (B)–(D) Comparison of *Bifidobacterium* abundance in the maternal gut, breast milk, and infant gut between the EB group and the MB group. *P*-values were calculated using the edgeR method. (E) Heatmap analysis of the breast milk microbiota in the EB and MB groups. A heatmap was constructed based on Bray–Curtis distances between samples.

To evaluate the specific composition of microbiota genera, we examined the top 10 most abundant genera for each sample (Figures 3, S3C and Tables S3–S5). The results revealed that *Faecalibacterium* was the dominant genera in the maternal gut, while *Lactobacillus* was dominant in the breast milk for the MNS and EB samples and Fusobacterium was dominant in the breast milk of the MB samples. The gut-derived *Escherichia-Shigella* (*Escherichia coli-Shigella* genus) and the strict anaerobic *Bacteroides* genera were observed in both the maternal gut microbiota (6.77% vs. 8.08%) and the breast milk (2.89% vs. 0.67%) in the MNS group. For both the mother’s stool samples and the breast milk samples, the MNS and EB groups were most similar to each other in terms of the distributions, each showing some differences relative to the MB group. Specifically, the genera *Klebsiella*, *Streptococcus* and *Clostridium* *sensu stricto1* were dominant in the MNS and EB groups in the gut, while *Lactobacillus* and *Klebsiella* were dominant in the MNS and EB groups in the breast milk. Compared to the MNS and EB samples, the MB samples presented a greater abundance of *Faecalibacterium* in both the gut and the breast milk and a greater abundance of both *Fusobacterium* and *Bifidobacterium* in the breast milk (*P* < 0.0001). These differences were less obvious in the infant stool samples and suggest that factors such as the frequency and duration of infant sucking may exert a reverse influence on the composition of the milk microbiota, resulting in *Bifidobacterium* not being a dominant genus.

Further comparison of the abundance of *Bifidobacterium* between the EB and MB groups showed no significant differences for the maternal gut samples (*P* = 0.5113), though the abundance in breast milk was lower in the EB group compared to the MB group, and there was greater variability in *Bifidobacterium* abundance in the EB group compared to the MB group (Figure 3). Comparison of 17 genera (relative abundances > 1%) in the mother stool, breast milk, and infant stool between the EB and MB group was shown in Figure S4 and Table S6. *Escherichia-Shigella* and *Fusobacterium* presented trends consistent with those of *Bifidobacterium.*

A heatmap based on sample-dependent Bray‒Curtis distances (Figure 3) revealed significant differences in the breast milk microbiota composition between the EB and MB groups. Another heatmap derived from the UniFrac distance was presented in Supplementary Figure S3D, which exhibited a consistent trend with the Bray‒Curtis heatmap. These results suggest that the feeding method influences both the abundance and composition of the breast milk microbiota.

### Traceability analyses of samples from different sources

**Figure 4.**
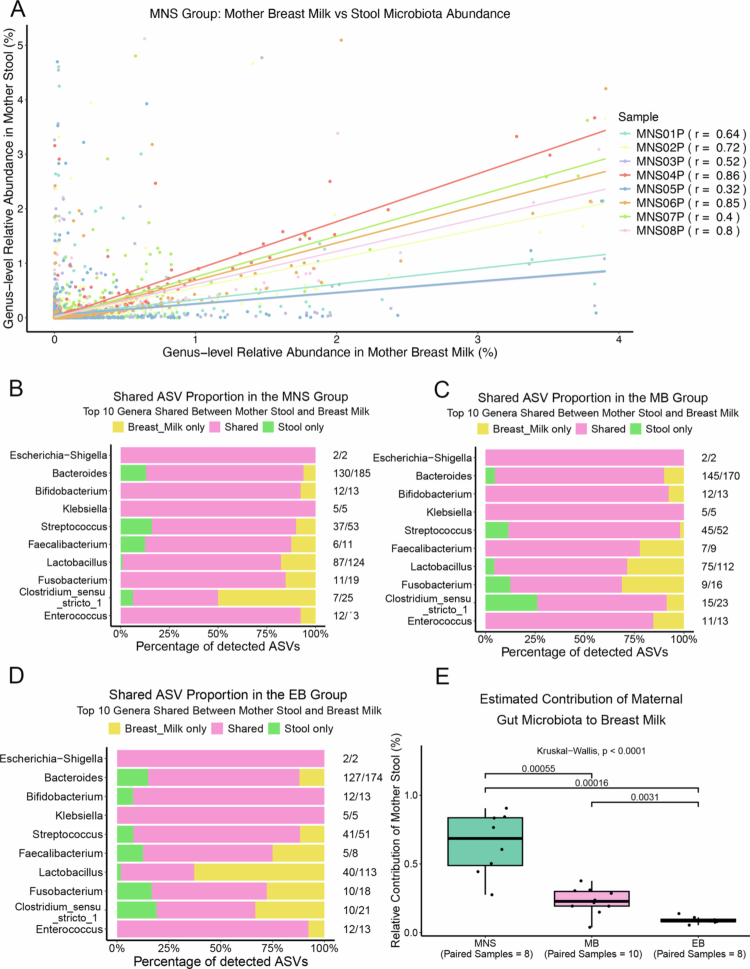
Breast milk–maternal gut microbiome traceback analysis. (A) A composite graph of the fitting curves for the breast milk–maternal gut microbiome correlation analysis in the MNS group. The Pearson correlation between the colostrum and maternal gut microbiomes for each mother‒infant pair in the MNS group was calculated at the genus level based on abundance. The results demonstrate a significant positive correlation at the genus level. (B)–(D) Bar charts showing the proportional composition of the microbiome originating from the breast milk of the MNS, EB, and MB groups, respectively, compared to the maternal gut microbiome. The top 10 ASVs (amplifier sequence variants) with the highest abundance in each group are shown. Each bar represents the proportion of ASVs from different sources within the total number of ASVs for each genus. Yellow indicates ASVs detected only in the breast milk, green indicates ASVs detected solely in the maternal feces, and pink indicates ASVs detected in both the breast milk and maternal feces. The numbers on the right represent the ratio of the total number of ASVs annotated to that genus to the total number of ASVs identified. (E) Analysis of the differences in the proportion of the breast milk microbiomes originating from the maternal gut microbiome of the MNS, MB and EB groups based on the R FEAST tracing method. Differences among multiple groups were assessed by the Kruskal‒Wallis test, followed by pairwise comparisons with the Wilcoxon rank‒sum test. (MNS: mother-newborn separated, EB: exclusive breastfeeding, MB: mixed breastfeeding).

To evaluate whether there is an association in the microbiome in different sources (maternal gut, breast milk, and infant gut) from the same mother‒infant pair, we performed Pearson correlation analysis. First, we compared the breast milk and maternal gut microbiota of 8 pairs in the MNS group. The resulting fitted curve indicated a significant positive correlation between the colostrum and maternal gut microbiota in the MNS group (Figure 4). This result suggests that the microbiota in breast milk may originate from the maternal gut microbiota. Next, we assessed the vertical strain sharing between the breast milk and the mother’s stool. Strain sharing was detected among the proportions of ASV originated in the MNS, MB and EB group from the top 10 most abundant genera (Figure 4). All 10 genera were shared between breast milk and maternal stool. These results are consistent with the possibility that the breast milk microbiota is derived from the mother's intestinal flora.

For additional insight into the cross-sample correlations, we applied the R FEAST method, a one-to-one strict paired traceability analysis of the breast milk-mother gut microbiota from the MNS, EB, and MB groups at the same lactation stage. The results showed that, in the MNS group, the average proportion of the breast milk microbiota originating from the mother's gut microbiota was as high as 67.9% (95% CI: 0.492–0.866) for 8 pairs (Figures 4 and S5A). The average proportion of maternal gut microbiota traced to breast milk in the MB group (10 pairs) was 21.1% (95% CI: 0.168–0.254, Figures 4 and S5B), while the corresponding proportion in the EB group (8 pairs) was 9% (95% CI: 0.07–0.109, Figures 4 and S5C). Additionally, we conducted a multivariate regression analysis on confounding factors using information from questionnaires. The results indicated that all variables, including maternal dietary, delivery mode, antibiotic use, infant gender and infant weight, did not significantly influence our traceability analysis results (Supplementary Table S7).

Taken together, the above results indicate that there are significant differences in the proportion of maternal milk microbiota derived from the intestinal microbiota among the MNS, MB, and EB groups. In the MNS group, where the mother’s breast was not sucked by the infant, the milk microbiota predominantly originated from the maternal gut. In the MB group, where infants receive both breast milk and formula, the milk microbiota is influenced by factors such as prebiotics in the formula and bacterial contamination during feeding, leading to a lower proportion of microbiota derived from the mother’s gut. In contrast, the EB group presented the lowest proportion of milk microbiota derived from the maternal gut, suggesting that the composition and abundance of the milk microbiota are influenced not only by the maternal gut microbiota but also by various factors, such as the frequency and duration of neonatal sucking (i.e., feeding mode).

### Development analysis of the infant gut microbiota

**Figure 5.**
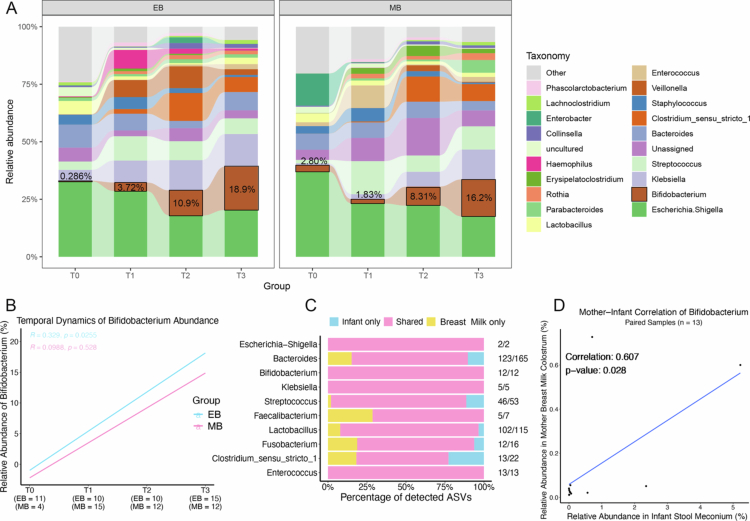
Development of the infant gut microbiota and *Bifidobacterium* within one month after birth. (A) The impact plot of infant gut microbiome development within one month under different feeding methods. The impact plot was generated using the R package ggalluvial (v0.12.5) to display the relative abundance changes of the top 20 genera (based on abundance at the genus level) and the remaining genera (referred to as “Other”) across different groups and time points. (B) The correlation trends of *Bifidobacterium* in the intestinal microbiota of infants in the EB and MB groups over time. The figure presents the Spearman correlation and *p*-values for *Bifidobacterium* across the four time points. (C) The bar chart shows the proportional origins of the infant gut microbiota and human milk microbiota across the four stages (T0–T3) in the EB group. The chart depicts the source proportions of amplicon sequence variants (ASVs) belonging to the top 10 most abundant genera. Each bar represents the proportion of ASVs from different sources relative to the total ASVs for the corresponding genus. Yellow indicates ASVs detected exclusively in human milk, blue represents ASVs detected exclusively in infant feces, and pink denotes ASVs identified in both human milk and infant feces. The numbers on the right side indicate the ratio of ASVs identified for each genus to the total number of ASVs annotated to that genus. (D) The correlation between *Bifidobacterium* in the meconium (T0) of infants and colostrum (T1) from EB was analyzed. A Spearman correlation coefficient of 0.607, with a *p*-value < 0.05, indicates a significant positive correlation.

To present the temporal dynamic changes in core microbes across different groups, a heatmap of key microbes (with a relative abundance greater than 1%) was generated to show the relative abundance changes in microbes caused by the “source-lactating stage” in the MNT group, which presented differences in transmission trends among different microbes (supplementary Figure S5E). To further investigate factors that contribute to the development of the infant gut microbiota, we generated impact plots for the EB and MB groups. The common dominant genera in these groups, including *Escherichia-Shigella* (comprising *Escherichia coli* and *Shigella*), *Bifidobacterium*, *Klebsiella*, *Streptococcus*, *Bacteroides*, and *Clostridium sensu stricto1* (Figures 5 and S6A), as well as their relative abundance, were found to fluctuate with the different lactation stages, which is consistent with existing research findings.^19^ The most significant variation was observed in *Bifidobacterium*, which exhibited a clear increase in abundance over time. Interestingly, there were marked differences in the developmental patterns of the gut microbiota between the EB and MB groups. For the EB group infants, *Escherichia-Shigella* and *Bacteroides* were enriched at birth. As lactation stages progressed, *Bifidobacterium* and *Klebsiella* gradually dominated in terms of abundance, while for the infants in the MB group, *Escherichia-Shigella* and *Enterobacter* were enriched at birth, followed by a gradual dominance of *Bifidobacterium*, *Klebsiella*, and *Streptococcus* as lactation stages advanced (Figure 5).

The correlation trend of *Bifidobacterium* in the infant gut over time for the EB and MB groups is further detailed in Figure 5. Although the abundance of *Bifidobacterium* increased over time in both the EB and MB groups, the slope for the EB group was steeper, indicating a stronger correlation between *Bifidobacterium* and time in the EB group, with a corresponding *p*-value < 0.05. In contrast, the correlation of *Bifidobacterium* over time in the MB group did not reach statistical significance (*p*-values > 0.05). These findings suggest that, compared to mixed feeding, exclusive breastfeeding is more beneficial for *Bifidobacterium*, potentially accelerating its growth, which indicates that exclusive breastfeeding plays a more beneficial role in the establishment of the infant gut microbiota.

For an additional understanding of the impact of breastfeeding on the correlation between the infant and maternal gut *Bifidobacterium*, we traced the source of the infant meconium microbiota through analysis of the meconium of infants in the EB group and the maternal colostrum. Using the R FEAST traceability method on 11 pairs of infant meconium and maternal colostrum samples, we assessed the contribution of each maternal milk sample to its corresponding infant fecal microbiota. The results suggest that 87.7% (95% CI: 0.767−0.987) of the microbiota in the meconium samples may have originated from the corresponding maternal colostrum (Figure S5D). Vertical strain sharing between the infant gut and EB milk was detected among each of the top 10 most abundant genera (Figure 5). These findings suggested that the infant gut microbiota was most likely derived from the mother's breast milk. To further explore the origins of the gut microbiota in infant meconium, a correlation analysis was performed on 17 genera (abundance > 1%) in the EB group between infant meconium (T0) and maternal colostrum (T1). The results with significant differences were illustrated in Supplementary Figure S6B–D. A positive correlation was observed between the *Bifidobacteria* in the infant's meconium and those in the maternal milk (Figure 5) (*p* = 0.028). These results support the hypothesis that bacterial transmission from breast milk to the infant gut occurs vertically.^20^

## Discussion

The acquisition and development of the infant microbiome are critical for establishing healthy host‒microbiome symbioses. Studies have highlighted the critical role of the mother-derived infant gut microbiota^3^. Mother–infant microbial transmission occurs from the beginning of pregnancy to well after delivery. This process exhibits distinct patterns and locations within the body, influenced by a variety of intrinsic and extrinsic factors.^21^ The maternal microbiota (derived from the intestine during vaginal delivery and from breast milk during feeding) seeds the infant intestine first, thus influencing and selecting subsequent microbiota and leaving a footprint that can be detected and even continue into adulthood.^1^ While the importance of the host‒microbiome interplay is well established, the mechanisms underlying the acquisition and development of the infant gut microbiome during early life remain largely unexplored.

In this study, we characterized the colonization and developmental patterns of the gut microbiota in healthy Chinese infants during early life, including the abundance, diversity, and correlations with the maternal gut and breast milk at different stages postpartum. Additionally, we conducted trace-back analysis of the microbiota in breast milk and the infant gut to explore the link between maternal-infant transmission link between maternal gut microbiota, breast milk microbiota, and infant gut microbiota in early human life. This approach provides a comprehensive perspective on the role of the maternal microbiota in infant growth, maturation, and development, while also offering evidence for the gut-breastmilk pathway hypothesis of the breast milk microbiota.

In our diversity analysis, the MNS group showed lower alpha diversity than the MB and EB groups in the maternal gut microbiota during the T1 phase, i.e., the colostrum period (within 5 d postpartum). As lactation progressed, no significant differences in the alpha and beta diversity of the gut microbiota were observed between EB and MB mothers, regardless of whether the infants were exclusively breastfed. Previous studies have shown that with the development of the fetus in the third trimester, the total load of maternal gut microbiota increases, and the beta diversity among pregnant individuals increases, whereas individual richness and evenness (alpha diversity) decrease.^22^^,^^23^ After delivery, the microbiota of the maternal gut retains the same patterns observed in the third trimester for more than 1 month postpartum.^8^^,^^23^^,^^24^ Therefore, our results are consistent with the trends in pregnancy.

Although our study employed rigorous sterile protocols to minimize environmental interference, there remains a theoretical risk that background contaminants could affect the results. This is particularly pertinent for low-biomass environments, such as breast milk samples, which are vulnerable to external contamination.^25^^,^^26^ Therefore, to mitigate potential artifacts arising from contamination or stochastic noise, we focused our analysis on highly abundant microbial genera. Specifically, we constrained our core analyses to bacterial genera with relative abundances exceeding 1% or prioritized the top 10−20 most abundant genera within each sample. This approach reduces the risk of overinterpreting low-abundance signals, which are more susceptible to contamination and often difficult to distinguish confidently from genuine biological variation.^27^ Regarding the composition of the dominant bacterial genera (i.e., the top 10 taxa by relative abundance) in breast milk, we found that *Lactobacillus* was more prevalent in the MNS and EB groups compared to the MB group (Figure 3). Additionally, the breast milk microbiota was enriched with gut-derived genera such as *Escherichia-Shigella* (*Escherichia* and *Shigella*), as well as strict anaerobes like *Bacteroides*, *Bifidobacterium*, and others. These findings directly support the gut‒breast translocation pathway hypothesis. Although some studies have shown that the composition of the breast milk microbiota undergoes substantial changes across different stages of lactation^28^, research investigating the microbiota of colostrum, milk at one month postpartum, and milk at six months postpartum has shown that colostrum presents greater microbial diversity than mature milk.^29^ However, the results of this study indicate that the diversity of the breast milk microbiota is not associated with the stage of lactation, suggesting that colostrum already contains a microbiota as rich as that found in mature milk, which is likely to facilitate the colonization of the infant's gut with the initial microbial community.

From the first to the third trimester, profound alterations have been observed in the guts of pregnant women, where butyrate-producing bacteria, such as *Faecalibacterium*, exhibit a significant decrease, while *Proteobacteria* and some lactic acid-producing bacteria, such as *Lactobacillus* and *Bifidobacteria*, are highly increased.^23^^,^^30^ Furthermore, previous studies have revealed the expansion of members of *Enterobacteriaceae* and *Bifidobacterium* in the mother’s stool in the third trimester.^8^^,^^23^^,^^24^ Our results (Figures 3 and S3C and Tables S3–S5) demonstrated that these bacteria might colonize the infant gut early, indicating potential transmission from the maternal gut to the neonatal gut. The gut microbiota of EB mothers included *Escherichia-Shigella* (12.80%) and *Bifidobacterium* (6.66%). The relative abundance of *Escherichia-Shigella* was greater in meconium (32.75%) compared to colostrum (28.57%), while *Bifidobacterium* showed a higher proportion in colostrum (3.72%) compared to meconium (0.29%). These results suggest that changes in the maternal gut microbiota during late pregnancy and postpartum likely facilitate the transfer of the first dominant microbiota genera from the maternal gut to the infant's gut, with the mediating factor being the breast milk microbiota. The vertical transmission of microbiota from breast milk and the horizontal transmission between non-kin through social interactions and shared environments have previously been shown to contribute to the colonization and establishment of the infant microbiota after birth.^8^

To explore the vertical sharing of microbiota, we conducted correlation analysis of the breast milk and maternal gut microbiota, which revealed positive correlations in all 8 mother‒infant pairs (0.19 ≤ *r* ≤ 0.84). Furthermore, vertical strain sharing was detected among the top 10 most abundant genera originating from ASV in the MNS group. Additionally, using the R FEAST tracing method, which involves strict mother-breast milk-gut microbiota pairing, we found that 67.9% of the breast milk microbiota was traced back to the maternal gut microbiota. The MNS analysis results strongly support the hypothesis that not all of the breast milk microbiota originates from external contamination and that a significant proportion is likely derived from the maternal gut. This serves as compelling evidence for the gut‒breast pathway hypothesis, whereby maternal intestinal bacteria migrate to the mammary glands via an endogenous cellular route during pregnancy and lactation.^31^

Our evidence suggests that the breast milk microbiota, in addition to being highly likely to originate from the maternal gut microbiota, is significantly influenced by the feeding method. The proportion of strictly gut-derived breast milk microbiota was significantly lower in the EB group compared to the MB group (*P* = 0.0031), and even more so compared to the MNS group (*P* = 0.00016). The underlying reason for this may be that infant suckling creates a negative pressure environment, and the oral microbiota of the infant and the breast milk microbiota have dynamic, reciprocal influences. The longer the infant suckles and the more frequently they do so, the more significant the impact on the breast milk microbiota. Consistently, the breast milk of the EB group was enriched with pioneer colonizers that are also present in the infant’s oral microbiota, including *Lactobacillus* (which accounts for 13.61% of EB colostrum), *Streptococcus,*^32^ and *Prevotella.*^33^ These bacteria significantly influence the relative abundance of some dominant probiotic genera in breast milk, such as *Bifidobacterium*. On the other hand, the MB group of breast milk was enriched with *Fusobacterium* (14.93%), a genus that is unique to formula-fed infants, which in turn influences the breast milk microbiota of the MB group.

At present, two main hypotheses have been proposed regarding the origin of the breast milk microbiota: the conventional contamination hypothesis and the revolutionary entero-mammary pathway hypothesis.^34^ The hypothesis that the maternal gut microbiota may migrate to the mammary gland via immune cells (e.g., dendritic cells) is supported by several studies^35^^,^^36^ or the lymphatic circulation.^37^ Our findings suggest a dual origin for the breast milk microbiota, which is primarily seeded by the maternal gut but is subsequently modulated by the infant's oral microbiota during breastfeeding. Furthermore, we demonstrated that in exclusively breastfed infants, there exists a dynamic, reciprocal exchange between the infant's oral microbiota and the breast milk microbiota. Therefore, we propose that the origins of the breast milk microbiota may be integrated into a model encompassing both endogenous and exogenous pathways, specifically the “maternal gut (endogenous entero-mammary pathway)/infant suckling (exogenous contamination) - mammary gland - breast milk” transmission route.

Pregnancy and lactation represent unique physiological stages during which the regulation of the gut microbiota in women undergoes corresponding changes. Indeed, the altered gut microbiota may facilitate the metabolism of the host during pregnancy through enhancing glucose and fatty acid absorption, modulating fasting-induced adipocyte factor secretion, suppressing anabolic pathways and inducing catabolic pathways, and stimulating the immune system^22^. Therefore, the modulation of the maternal gut microbiota during pregnancy and lactation is likely to have a direct association with infant health. Breast milk not only provides essential nutrients for infants but also supplies hundreds of thousands of years of evolution, and the commensal microbiota, including more than 700 bacterial species at concentrations of approximately 1000 colony-forming units (CFUs)/ml, is also present in breast milk, which serves as pioneering colonizers of the infant gut,^38^^,^^39^ improves nutrient metabolism and absorption, and enhances the development of the immune system and intestinal barrier and the gut–brain axis in the infant.^39^ Our previous findings^40^ and the current study confirm this conclusion, demonstrating that breast milk is abundant in bacteria, with a high relative abundance of bacterial genera.

A prior study demonstrated that the maternal fecal microbiota accounts for the highest proportion of the infant gut microbiota, comprising 22.1% of its total abundance.^3^ However, human milk consumption introduces the infant to hundreds of phylotypes of bacteria that have a direct route to the gastrointestinal tract. Consequently, human milk-associated microbes are among the first to colonize the infant gut. It is hypothesized that these factors may help to shape infant health outcomes in the short- and long-term. The short-term outcomes may include fewer cases of diarrhea, pneumonia, otitis media, atopic dermatitis, and sudden infant death syndrome.^41^^,^^42^ The long-term outcomes may include fewer cases of risk of food allergy, type 2 diabetes, obesity and leukemia.^43-45^ In addition, there may be beneficial effects on IQ and social behavior.^46^ Furthermore, there is a plethora of health benefits associated with breastfeeding and human milk for mothers, including a reduced risk of breast and ovarian cancer, hypertension, and type 2 diabetes.^47^ Accordingly, exclusive breastfeeding, especially longer than 2 months from birth, is associated with a more stable gut bacterial taxa composition.^48^ Substantial differences exist in the gut microbiota composition and metabolomic profiles between breastfed and exclusively formula-fed infants.^49^ Breastfed infants typically exhibit a microbial community characterized by lower alpha diversity and a predominance of *Bifidobacterium* species.^50^^,^^51^ In contrast, exclusively formula-fed infants tend to demonstrate greater bacterial diversity and a more mature microbial architecture.^52^ Furthermore, comparative analyses have revealed significant divergence in the overall taxonomic composition of the gut microbiome depending on the feeding mode.^53^ Previous research has indicated that breastfeeding profoundly influences the structure and developmental trajectory of the infant gut microbiome, irrespective of whether it is provided exclusively or in combination with formula.^49^^,^^54^

Our results suggest that during the early establishment of the gut microbiota, the first microbial actors that render the gastro-intestinal environment fully anaerobic may be facultative anaerobes. These include bacteria such as *Enterobacteriaceae* (e.g., *Escherichia coli* and *Shigella*), *Streptococcus*, *Klebsiella*, *Bacteroides*, *Staphylococcus*, and *Lactobacillus*. These microbiota dominate in the early days of an infant’s life and may deplete the oxygen in the infant gut, contribute to the formation of a strictly anaerobic environment in the gut^55^^,^^56^ and create a suitable environment with low redox potential for the growth of anaerobic bacteria.^2^ After the removal of oxygen, the infant intestine undergoes extensive colonization driven by strictly anaerobic bacterial taxa, such as those belonging to the genera *Bifidobacterium*, *Clostridium*, *Bacteroides* and *Ruminococcus.*^57^^,^^58^ Then, the infant continues to acquire microbes from distinct maternal sources after birth. Maternal gut strains are more persistent in the infant gut and are ecologically better adapted than those acquired from other sources. Some bacteria, such as strains from the mother's vaginal, oral, and skin microbiota, transiently colonize the infant gut. These bacteria may not be well-adapted or suitable for colonization of the infant's lower gastrointestinal tract, and as a result, they are easily lost or replaced.^3^ Furthermore, vertical mother-to-infant microbiota transmission is tightly regulated, and the microbes that colonize in early life are selected rather than allowing microbial acquisition by the infant to chance.^8^

In our study, *Bifidobacterium* was detected in the gut, though there were no differences in abundance over time. These findings strongly suggest that the composition of the maternal gut microbiota, as well as the specific probiotic genera, remains stable and is not influenced in reverse by the oral microbiota of the infant or the mammary gland microbiota during breastfeeding. To obtain a more detailed understanding of vertical transmission mechanisms, we performed a focused analysis of *Bifidobacterium* in the EB group. *Bifidobacterium*, one of the first colonizers, is the dominant bacterium in breastfed infants and has important effects on the development of the gut microbiota and subsequent physiological state and infant health.^59^ This trend showed a greater correlation with the lactation phase, indicating that exclusive breastfeeding accelerates the growth of *Bifidobacterium* in the infant gut, thereby promoting the establishment of a more stable gut microbiota. This phenomenon is closely linked to the over 200 oligosaccharides in breast milk that can be broken down and utilized by the infant's gut bacteria, particularly *Bifidobacterium*^3^. The food of bifidobacteria is human milk oligosaccharides (HMOs), the third most abundant ingredient in human breast milk and a prebiotic.^60^ HMOs promote a gut microbiome rich in bifidobacteria. Some members of the *Bifidobacterium* genus, including *Bifidobacterium bifidum* and *B. longum subsp. infantis*, can metabolize HMOs for growth and proliferation. These two species dominate through inhibitory effects, whereby the first species depletes resources for later arrivals.^61-63^ In addition to promoting a *Bifidobacterium*-rich gut microbiome, HMOs also improve the gastrointestinal barrier. They protect against infection, strengthen the epithelial barrier and create immunomodulatory metabolites.^60^ The exposure of breastfed infants to bacterial richness in milk may be an additional factor contributing to the differential fecal microbiota between breastfed and formula-fed infants.^64^ Our analysis revealed a positive correlation with the number of ASVs shared at a 100% ratio. This provides direct evidence that *Bifidobacterium* is vertically transferred from the maternal microbiota to the infant gut microbiota, serving as an important marker for maternal–infant microbiome inheritance. *Bifidobacterium*, an anaerobic bacterium, has been theorized to facilitate the transmission of the maternal gut-breast milk-infant intestine through the endogenous entero-mammary pathway. Bacterial translocation from the intestine to the mammary glands is believed to occur primarily via gut-associated lymphoid tissues,^37^^,^^65^^,^^66^ involving key immune cells such as dendritic cells and macrophages,^67^ which may act as carriers of bacteria from the maternal gut to the mammary gland.^68^^,^^69^ A preceding study on non-pathogenic microorganisms indicated that dendritic cells may express tight junction proteins, which results in the opening of tight junctions between epithelial cells, thereby enabling their penetration of the gut epithelial monolayers to capture bacteria.^67^ However, the specific transmission mechanism of *Bifidobacterium* still requires further experimental validation in our subsequent studies.

We also observed high shared proportions of *Escherichia coli–Shigella* (100%), *Klebsiella* (100%), *Enterococcus* (100%), *Lactobacillus* (89%), and *Streptococcus* (87%). Vertically transferred strains have been proven to be involved in several specific functional traits in infants, such as immune stimulation during the first week of life, which profoundly influences the health of the host in later life.^70^ Several of the bacteria ‘‘shared’’ between breastmilk and the infant gut are involved in either lactate production (such as *Bifidobacterium*, *Streptococcus* and *Lactobacillus*) or utilization (*Veillonella*). The copresence of lactate producers and utilizers in the infant gut may facilitate a trophic chain that avoids lactate build-up and its detrimental sequelae.^71^ Furthermore, *Bifidobacteria* produce acetate through the glycosylation fermentation of oligosaccharides, which, along with lactate, help maintain a lower intestinal pH, thereby preventing the colonization of opportunistic pathogens.^72^

Limitations of our study include the small sample size and uncontrolled dietary factors. As noted in previous research, this can limit the study's power and generalizability, potentially introducing biases or reducing the robustness of the findings. For instance, such constraints may restrict the ability to detect subtle effects or generalize our results to broader populations.^73^ Additionally, we did not impose strict dietary controls on the participants, which is one of the limitations of our study. Diets can introduce confounding variables, as variations in nutrient intake or food choices might influence the microbiome composition.^74^ Maternal diet directly affects breast milk bioactive components, including microRNAs, immune factors, oligosaccharides, and polyphenols, which in turn may modulate infant microbial communities and development.^75^ Despite these limitations, our analysis allowed us to identify potential sources and pathways of microbial transmission, which could inform future research on infant microbiome development. The necessity for future large-scale, multicenter studies and dietary monitoring for validation is indicated.

## Conclusion

As one of the most significant sources of the initial bacterial community, breast milk is the gold standard for nutritional sustenance from birth through the first few months of a newborn's growth and development. The World Health Organization recommends exclusive breastfeeding for the first six months of life.^76^ From an evolutionary perspective, the entero-mammary pathway theory and the composition of human milk are important because certain bacterial species undergo strong selection to initiate the colonization of the intestinal microbiota of the newborn for subsequent maturation ^77^

In this study, we identified very high microbial diversity in the pioneering infant gut microbiome even on the first day of life. Furthermore, we elucidated the temporal characteristics and the basis for vertical transmission of the microbiota from the maternal gut to breast milk and subsequently to the infant gut. This holistic perspective of the transmission of maternal gut and breast milk microbiota to the infant's gut provides us with more comprehensive and direct evidence of the pathway through which microbial heritage is passed from mother to child. Comprehensive insights into microbial transmission, colonization and succession from the aspects of mother and infant greatly promote the success of maternal and neonatal microbiome studies.

Moreover, considering the influence of the maternal gut microbiota on the breast milk microbiota, which ultimately affects the development of the infant's gut microbiota, ensuring the health of the mother's gut microbiota throughout the entire perinatal period is essential. This will better promote the establishment, development, and health of the infant's gut microbiota.

Although these results support the hypothesis of vertical transmission of microbiota from breast milk to the infant gut, the proportion and identity of shared microbes in these studies should be interpreted with some caution, as the maternal gut microbiota, the largest donor of the infant microbiota, is likely to play a dominant role. In the future, whole-genome analysis based on single nucleotide variants will enable the exploration of microbial transmission events, offering more precise evidence and deeper insights from the species level down to a high taxonomic resolution (strain level) for each individual. This approach will provide more accurate and profound insights, identify the most probable pathways of maximal microbiota transfer and delve into the mechanisms of maternal–infant microbial heritage and the interactions between the microbiota in the infant's gut.

## Supplementary Material

Supplementary material Supplementary Information.

Supplementary material Supplementary Information.

Supplementary material Supplementary Information.

Supplementary material Supplementary Information.

Supplementary material Supplementary Information.

Supplementary material Supplementary Information.

Supplementary material Supplementary Information.

Supplementary material Table S1. The demographics of the participants.

Supplementary material Supplementary Information.

Supplementary material Supplementary Information.

Supplementary material Table S7. Confounding factor analysis.

Supplementary material Supplementary Information.

Supplementary material Table S6. Comparison of 17 genera (relative abundance > 1%) in the mother stool, breast milk, and infant stool between the EB and MB groups.

## Data Availability

The data generated and analyzed in this study are not publicly available at this time owing to privacy protection. However, partial data may be shared upon reasonable request by contacting the corresponding author. The requests should be submitted to the corresponding authors for review and approval.

## References

[1] Yang B, Ding M, Chen Y, Han F, Yang C, Zhao J, Malard P, Stanton C, Ross RP, Zhang H. Development of gut microbiota and bifidobacterial communities of neonates in the first 6 weeks and their inheritance from mother. Gut Microbes. 2021;13:1–13. doi: 10.1080/19490976.2021.1908100. PMC8049200 33847206

[2] Velez-Ixta JM, Juarez-Castelan CJ, Ramirez-Sanchez D, Lazaro-Perez NDS, Castro-Arellano JJ, Romero-Maldonado S, Vélez-Ixta JM, Juárez-Castelán CJ, Ramírez-Sánchez D, Lázaro-Pérez NdS, et al. Post natal microbial and metabolite transmission: the path from mother to infant. Nutrients. 2024;16:1990. doi: 10.3390/nu16131990. 38999737 PMC11243545

[3] Ferretti P, Pasolli E, Tett A, Asnicar F, Gorfer V, Fedi S, Armanini F, Truong DT, Manara S, Zolfo M, et al. Mother-to-INFANT MIcrobial transmission from different body sites shapes the developing infant gut microbiome. Cell Host Microbe. 2018;24:133–145. e5. doi: 10.1016/j.chom.2018.06.005. 30001516 PMC6716579

[4] Feehley T, Plunkett CH, Bao R, Choi Hong SM, Culleen E, Belda-Ferre P, Campbell E, Aitoro R, Nocerino R, Paparo L, et al. Healthy infants harbor intestinal bacteria that protect against food allergy. Nat Med. 2019;25:448–453. doi: 10.1038/s41591-018-0324-z. 30643289 PMC6408964

[5] Vatanen T, Franzosa EA, Schwager R, Tripathi S, Arthur TD, Vehik K, Lernmark Å, Hagopian WA, Rewers MJ, She J, et al. The human gut microbiome in early-onset type 1 diabetes from the TEDDY study. Nature. 2018;562:589–594. doi: 10.1038/s41586-018-0620-2. 30356183 PMC6296767

[6] Tamburini S, Shen N, Wu HC, Clemente JC. The microbiome in early life: implications for health outcomes. Nat Med. 2016;22:713–722. doi: 10.1038/nm.4142. 27387886

[7] Rajilic-Stojanovic M, Heilig HG, Tims S, Zoetendal EG, de Vos WM. Long-term monitoring of the human intestinal microbiota composition. Environ Microbiol. 2012 10.1111/1462-2920.12023 23286720

[8] Wang S, Ryan CA, Boyaval P, Dempsey EM, Ross RP, Stanton C. Maternal vertical transmission affecting early-life microbiota development. Trends Microbiol. 2020;28:28–45. doi: 10.1016/j.tim.2019.07.010. 31492538

[9] Jost T, Lacroix C, Braegger C, Chassard C. Assessment of bacterial diversity in breast milk using culture-dependent and culture-independent approaches. Br J Nutr. 2013;110:1253–1262. doi: 10.1017/S0007114513000597. 23507238

[10] Murphy K, Curley D, O'Callaghan TF, O'Shea CA, Dempsey EM, O'Toole PW, O’Callaghan TF, O’Shea C, O’Toole PW, Ross RP, et al. The composition of human milk and infant faecal microbiota over the first three months of life: a pilot study. Sci Rep. 2017;7:40597. doi: 10.1038/srep40597. 28094284 PMC5240090

[11] Urbaniak C, Gloor GB, Brackstone M, Scott L, Tangney M, Reid G. The microbiota of breast tissue and its association with breast cancer. Appl Environ Microbiol. 2016;82:5039–5048. doi: 10.1128/AEM.01235-16. 27342554 PMC4968547

[12] Yang C, Xu ZX, Deng QC, Huang QD, Wang X, Huang FH. Beneficial effects of flaxseed polysaccharides on metabolic syndrome via gut microbiota in high-fat diet fed mice. Food Res Int. 2020;131:108994. doi: 10.1016/j.foodres.2020.108994. 32247451

[13] Sheng DD, Zhao SM, Gao L, Zheng HF, Liu WT, Hou J, Jin Y, Ye F, Li R, Zhang L, et al. BabaoDan attenuates high-fat diet-induced non-alcoholic fatty liver disease via activation of AMPK signaling. Cell Biosci. 2019;9:77. doi: 10.1186/s13578-019-0339-2. 31548878 PMC6751621

[14] Bolyen E, Rideout JR, Dillon MR, Bokulich N, Abnet CC, Al-Ghalith GA, Alexander H, Alm EJ, Arumugam M, Asnicar F, et al. Reproducible, interactive, scalable and extensible microbiome data science using QIIME 2. Nat Biotechnol. 2019;37:852–857. doi: 10.1038/s41587-019-0209-9. 31341288 PMC7015180

[15] Callahan BJ, McMurdie PJ, Rosen MJ, Han AW, Johnson AJA, Holmes SP. DADA2: High-resolution sample inference from illumina amplicon data. Nat Methods. 2016;13:581–583. doi: 10.1038/nmeth.3869. 27214047 PMC4927377

[16] Liu YX, Qin Y, Chen T, Lu MP, Qian XB, Guo XX, Bai Y. A practical guide to amplicon and metagenomic analysis of microbiome data. Protein Cell. 2021;12:315–330. doi: 10.1007/s13238-020-00724-8. 32394199 PMC8106563

[17] Lozupone CA, Hamady M, Kelley ST, Knight R. Quantitative and qualitative β diversity measures lead to different insights into factors that structure microbial communities. Appl Environ Microb. 2007;73:1576–1585. doi: 10.1128/AEM.01996-06. PMC1828774 17220268

[18] Shenhav L, Thompson M, Joseph TA, Briscoe L, Furman O, Bogumil D, Mizrahi I, Pe’er I, Halperin E. FEAST: fast expectation-maximization for microbial source tracking. Nat Methods. 2019;16:627–632. doi: 10.1038/s41592-019-0431-x. 31182859 PMC8535041

[19] Yang R, Gao R, Cui S, Zhong H, Zhang X, Chen Y, Wang J, Qin H. Dynamic signatures of gut microbiota and influences of delivery and feeding modes during the first 6 months of life. Physiol Genomics. 2019;51:368–378. doi: 10.1152/physiolgenomics.00026.2019. 31226006

[20] Differding MK, Mueller NT. Human milk bacteria: seeding the infant gut? Cell Host Microbe. 2020;28:151–153. doi: 10.1016/j.chom.2020.07.017. 32791106

[21] Xiao LW, Zhao FQ. Microbial transmission, colonisation and succession: from pregnancy to infancy. Gut. 2023;72:772–786. doi: 10.1136/gutjnl-2022-328970. 36720630 PMC10086306

[22] Collado MC, Isolauri E, Laitinen K, Salminen S. Distinct composition of gut microbiota during pregnancy in overweight and normal-weight women. Am J Clin Nutr. 2008;88:894–899. doi: 10.1093/ajcn/88.4.894. 18842773

[23] Koren O, Goodrich JK, Cullender TC, Spor A, Laitinen K, Backhed HK, Kling Bäckhed H, Gonzalez A, Werner JJ, Angenent LT, et al. Host remodeling of the gut microbiome and metabolic changes during pregnancy. Cell. 2012;150:470–480. doi: 10.1016/j.cell.2012.07.008. 22863002 PMC3505857

[24] DiGiulio DB, Callahan BJ, McMurdie PJ, Costello EK, Lyell DJ, Robaczewska A, Sun CL, Goltsman DSA, Wong RJ, Shaw G, et al. Temporal and spatial variation of the human microbiota during pregnancy. Proc Natl Acad Sci U S A. 2015;112:11060–11065. doi: 10.1073/pnas.1502875112. 26283357 PMC4568272

[25] Spreckels JE, Fernández-Pato A, Kruk M, Kurilshikov A, Garmaeva S, Sinha T, Ghosh H, Harmsen H, Fu J, Gacesa R, et al. Analysis of microbial composition and sharing in low-biomass human milk samples: a comparison of DNA isolation and sequencing techniques. ISME Commun. 2023;3:116. doi: 10.1038/s43705-023-00325-6. 37945978 PMC10636111

[26] Stinson LF, Ma J, Sindi AS, Geddes DT. Methodological approaches for studying the human milk microbiome. Nutr Rev. 2023;81:705–715. doi: 10.1093/nutrit/nuac082. 36130405

[27] Dyrhovden R, Rippin M, Ovrebo KK, Nygaard RM, Ulvestad E, Kommedal O. Managing contamination and diverse bacterial loads in 16S rRNA deep sequencing of clinical samples: implications of the law of small numbers. mBio. 2021;12:e0059821. doi:10.1128/mBio.00598-21. 34101489 PMC8262989

[28] Lopez Leyva L, Brereton NJB, Koski KG. Emerging frontiers in human milk microbiome research and suggested primers for 16S rRNA gene analysis. Comput Struct Biotechnol J. 2021;19:121–133. doi: 10.1016/j.csbj.2020.11.057. 33425245 PMC7770459

[29] Cabrera-Rubio R, Collado MC, Laitinen K, Salminen S, Isolauri E, Mira A. The human milk microbiome changes over lactation and is shaped by maternal weight and mode of delivery. Am J Clin Nutr. 2012;96:544–551. doi: 10.3945/ajcn.112.037382. 22836031

[30] Nuriel-Ohayon M, Neuman H, Ziv O, Belogolovski A, Barsheshet Y, Bloch N, Uzan A, Lahav R, Peretz A, Frishman S, et al. Progesterone increases bifidobacterium relative abundance during late pregnancy. Cell Rep. 2019;27:730–736. e3. 10.1016/j.celrep.2019.03.075. 30995472

[31] Gomez-Gallego C, Garcia-Mantrana I, Salminen S, Collado MC. The human milk microbiome and factors influencing its composition and activity. Semin Fetal Neonatal Med. 2016;21:400–405. doi: 10.1016/j.siny.2016.05.003. 27286644

[32] Williams JE, Carrothers JM, Lackey KA, Beatty NF, Brooker SL, Peterson HK, Steinkamp KM, York MA, Shafii B, Price WJ, et al. Strong multivariate relations exist among milk, oral, and fecal microbiomes in mother-infant dyads during the first six months postpartum. J Nutr. 2019;149:902–914. doi: 10.1093/jn/nxy299. 31063198 PMC6543206

[33] Jost T, Lacroix C, Braegger CP, Rochat F, Chassard C. Vertical mother-neonate transfer of maternal gut bacteria via breastfeeding. Environ Microbiol. 2014;16:2891–2904. doi: 10.1111/1462-2920.12238. 24033881

[34] Lif Holgerson P, Esberg A, West CE, Johansson I. The breast milk and childhood gastrointestinal microbiotas and disease outcomes: a longitudinal study. Pediatr Res. 2023;93:570–578. doi: 10.1038/s41390-022-02328-w. 36216869 PMC9988688

[35] Dombrowska-Pali A, Wiktorczyk-Kapischke N, Chrustek A, Olszewska-Słonina D, Gospodarek-Komkowska E, Socha MW. Human milk microbiome-a review of scientific reports. Nutrients. 2024;16:1420. doi: 10.3390/nu16101420. 38794658 PMC11124344

[36] Lyons KE, Ryan CA, Dempsey EM, Ross RP, Stanton C. Breast milk, a source of beneficial microbes and associated benefits for infant health. Nutrients. 2020;12:1039. doi: 10.3390/nu12041039. 32283875 PMC7231147

[37] Yu J, Li W, Xu R, Liu X, Gao G, Kwok LY, Chen Y, Sun Z, Zhang H. Probio-M9, a breast milk-originated probiotic, alleviates mastitis and enhances antibiotic efficacy: insights into the gut-mammary axis. Imeta. 2024;3:e224. doi: 10.1002/imt2.224. 39135694 PMC11316926

[38] Heikkila MP, Saris PE. Inhibition of Staphylococcus aureus by the commensal bacteria of human milk. J Appl Microbiol. 2003;95:471–478. doi: 10.1046/j.1365-2672.2003.02002.x. 12911694

[39] Le Doare K, Holder B, Bassett A, Pannaraj PS. Mother's Milk: a purposeful contribution to the development of the infant microbiota and immunity. Front Immunol. 2018;9:361. doi: 10.3389/fimmu.2018.00361. 29599768 PMC5863526

[40] Du YL, Qiu Q, Cheng J, Huang ZL, Xie RX, Wang L, Han Z, Jin G. Comparative study on the microbiota of colostrum and nipple skin from lactating mothers separated from their newborn at birth in China. Front Microbiol. 2022;13. doi: 10.3389/fmicb.2022.932495. PMC9574262 36262322

[41] Christensen N, Bruun S, Søndergaard J, Christesen HT, Fisker N, Zachariassen G, Sangild PT, Husby S. Breastfeeding and infections in early childhood: a cohort study. Pediatrics. 2020;146. doi: 10.1542/peds.2019-1892. 33097658

[42] Sankar MJ, Sinha B, Chowdhury R, Bhandari N, Taneja S, Martines J, Bahl R. Optimal breastfeeding practices and infant and child mortality: a systematic review and meta-analysis. Acta Paediatr. 2015;104:3–13. doi: 10.1111/apa.13147. 26249674

[43] Matsumoto N, Yorifuji T, Nakamura K, Ikeda M, Tsukahara H, Doi H. Breastfeeding and risk of food allergy: a nationwide birth cohort in Japan. Allergol Int. 2020;69:91–97. doi: 10.1016/j.alit.2019.08.007. 31540813

[44] Horta BL, Loret de Mola C, Victora CG. Long-term consequences of breastfeeding on cholesterol, obesity, systolic blood pressure and type 2 diabetes: a systematic review and meta-analysis. Acta Paediatr. 2015;104:30–37. doi: 10.1111/apa.13133. 26192560

[45] Schraw JM, Bailey HD, Bonaventure A, Mora AM, Roman E, Mueller BA, Clavel J, Petridou ET, Karalexi M, Ntzani E, et al. Infant feeding practices and childhood acute leukemia: findings from the childhood cancer & leukemia international consortium. Int J Cancer. 2022;151:1013–1023. doi: 10.1002/ijc.34062. 35532209 PMC12893794

[46] Plunkett BA, Mele L, Casey BM, Varner MW, Sorokin Y, Reddy UM, Wapner RJ, Thorp JM, Saade GR, Tita AT, et al.. Association of breastfeeding and child IQ score at age 5 years. Obstet Gynecol. 2021;137:561–570. doi: 10.1097/AOG.0000000000004314. 33706345 PMC8104129

[47] Tschiderer L, Seekircher L, Kunutsor SK, Peters SAE, O'Keeffe LM, Willeit P. Breastfeeding is associated with a reduced maternal cardiovascular risk: systematic review and meta-analysis involving data from 8 studies and 1 192 700 Parous Women. J Am Heart Assoc. 2022;11:e022746. doi: 10.1161/JAHA.121.022746. 35014854 PMC9238515

[48] Jennewein MF, Abu-Raya B, Jiang Y, Alter G, Marchant A. Transfer of maternal immunity and programming of the newborn immune system. Semin Immunopathol. 2017;39:605–613. doi: 10.1007/s00281-017-0653-x. 28971246

[49] Stewart CJ, Ajami NJ, O'Brien JL, Hutchinson DS, Smith DP, Wong MC, O’Brien JL, Ross MC, Lloyd RE, Doddapaneni H, et al. Temporal development of the gut microbiome in early childhood from the TEDDY study. Nature. 2018;562:583–588. doi: 10.1038/s41586-018-0617-x. 30356187 PMC6415775

[50] Milani C, Duranti S, Bottacini F, Casey E, Turroni F, Mahony J, Belzer C, Delgado Palacio S, Arboleya Montes S, Mancabelli L, et al. The first microbial colonizers of the human gut: composition, activities, and health implications of the infant gut microbiota. Microbiol Mol Biol Rev. 2017;81. doi: 10.1128/MMBR.00036-17. PMC5706746 29118049

[51] Lawson MAE, O'Neill IJ, Kujawska M, Gowrinadh Javvadi S, Wijeyesekera A, Flegg Z, O’Neill IJ, Chalklen L, Hall LJ. Breast milk-derived human milk oligosaccharides promote Bifidobacterium interactions within a single ecosystem. Isme J. 2020;14:635–648. doi: 10.1038/s41396-019-0553-2. 31740752 PMC6976680

[52] Baumann-Dudenhoeffer AM, D'Souza AW, Tarr PI, Warner BB, Dantas G. Infant diet and maternal gestational weight gain predict early metabolic maturation of gut microbiomes. Nat Med. 2018;24:1822–1829. doi: 10.1038/s41591-018-0216-2. 30374198 PMC6294307

[53] Heppner N, Reitmeier S, Heddes M, Merino MV, Schwartz L, Dietrich A, List M, Gigl M, Meng C, van der Veen DR, et al. Diurnal rhythmicity of infant fecal microbiota and metabolites: a randomized controlled interventional trial with infant formula. Cell Host Microbe. 2024;32:573–587. e5. doi:10.1016/j.chom.2024.02.015. 38569545

[54] Xiao L, Zhao F. Microbial transmission, colonisation and succession: from pregnancy to infancy. Gut. 2023;72:772–786. doi: 10.1136/gutjnl-2022-328970. 36720630 PMC10086306

[55] Mueller NT, Bakacs E, Combellick J, Grigoryan Z, Dominguez-Bello MG. The infant microbiome development: mom matters. Trends Mol Med. 2015;21:109–117. doi: 10.1016/j.molmed.2014.12.002. 25578246 PMC4464665

[56] Mackie RI, Sghir A, Gaskins HR. Developmental microbial ecology of the neonatal gastrointestinal tract. Am J Clin Nutr. 1999;69:1035S–1045S. doi: 10.1093/ajcn/69.5.1035s. 10232646

[57] Jimenez E, Marin ML, Martin R, Odriozola JM, Olivares M, Xaus J, Jiménez E, Marín ML, Martín R, Fernández L, et al. Is meconium from healthy newborns actually sterile? Res Microbiol. 2008;159:187–193. doi: 10.1016/j.resmic.2007.12.007. 18281199

[58] Matamoros S, Gras-Leguen C, Le Vacon F, Potel G, de La Cochetiere MF. Development of intestinal microbiota in infants and its impact on health. Trends Microbiol. 2013;21:167–173. doi: 10.1016/j.tim.2012.12.001. 23332725

[59] Turroni F, Peano C, Pass DA, Foroni E, Severgnini M, Claesson MJ, Kerr C, Hourihane J, Murray D, Fuligni F, et al. Diversity of bifidobacteria within the infant gut microbiota. PLoS One. 2012;7:e36957. doi: 10.1371/journal.pone.0036957. 22606315 PMC3350489

[60] Dinleyici M, Barbieur J, Dinleyici EC, Vandenplas Y. Functional effects of human milk oligosaccharides (HMOs). Gut Microbes. 2023;15:2186115. doi: 10.1080/19490976.2023.2186115. 36929926 PMC10026937

[61] Zhang B, Li LQ, Liu F, Wu JY. Human milk oligosaccharides and infant gut microbiota: molecular structures, utilization strategies and immune function. Carbohydr Polym. 2022;276:118738. doi: 10.1016/j.carbpol.2021.118738. 34823774

[62] Lin C, Lin Y, Zhang H, Wang G, Zhao J, Zhang H, Chen W. Intestinal 'infant-type' bifidobacteria mediate immune system development in the first 1000 days of life. Nutrients. 2022;14:1498. doi: 10.3390/nu14071498. 35406110 PMC9002861

[63] Ojima MN, Jiang L, Arzamasov AA, Yoshida K, Odamaki T, Xiao J, Nakajima A, Kitaoka M, Hirose J, Urashima T, et al. Priority effects shape the structure of infant-type Bifidobacterium communities on human milk oligosaccharides. ISME J. 2022;16:2265–2279. doi: 10.1038/s41396-022-01270-3. 35768643 PMC9381805

[64] Yoshioka H, Iseki K, Fujita K. Development and differences of intestinal flora in the neonatal period in breast-fed and bottle-fed infants. Pediatrics. 1983;72:317–321. doi: 10.1542/peds.72.3.317. 6412205

[65] Vazquez-Torres A, Jones-Carson J, Bäumler AJ, Falkow S, Valdivia R, Brown W, Le M, Berggren R, Parks WT, Fang FC. Extraintestinal dissemination of Salmonella by CD18-expressing phagocytes. Nature. 1999;401:804–808. doi: 10.1038/44593. 10548107

[66] Qutaishat SS, Stemper ME, Spencer SK, Borchardt MA, Opitz JC, Monson TA, Anderson JL, Ellingson JLE. Transmission of Salmonella enterica serotype typhimurium DT104 to infants through mother's breast milk. Pediatrics. 2003;111(6):1442–1446. doi: 10.1542/peds.111.6.1442. 12777569

[67] Rescigno M, Urbano M, Valzasina B, Francolini M, Rotta G, Bonasio R, Granucci F, Kraehenbuhl J, Ricciardi-Castagnoli P. Dendritic cells express tight junction proteins and penetrate gut epithelial monolayers to sample bacteria. Nat Immunol. 2001;2:361–367. doi: 10.1038/86373. 11276208

[68] Perez PF, Doré J, Leclerc M, Levenez F, Benyacoub J, Serrant P, Doré J, Segura-Roggero I, Schiffrin EJ, Donnet-Hughes A. Bacterial imprinting of the neonatal immune system: lessons from maternal cells? Pediatrics. 2007;119:e724–e732. doi: 10.1542/peds.2006-1649. 17332189

[69] Fernandez L, Marin ML, Langa S, Martin R, Reviriego C, Fernandez A, Olivares M, Xaus J, Rodriguez JM. A novel genetic label for detection of specific probiotic lactic acid bacteria. Food Sci Technol Int. 2004;10:101–108. doi: 10.1177/1082013204043761.

[70] Wampach L, Heintz-Buschart A, Fritz JV, Ramiro-Garcia J, Habier J, Herold M, Narayanasamy S, Kaysen A, Hogan AH, Bindl L, et al. Birth mode is associated with earliest strain-conferred gut microbiome functions and immunostimulatory potential. Nat Commun. 2018;9:5091. doi: 10.1038/s41467-018-07631-x. 30504906 PMC6269548

[71] Pham VT, Lacroix C, Braegger CP, Chassard C. Lactate-utilizing community is associated with gut microbiota dysbiosis in colicky infants. Sci Rep. 2017;7:11176. doi: 10.1038/s41598-017-11509-1. 28894218 PMC5593888

[72] Thomson P, Medina DA, Garrido D. Human milk oligosaccharides and infant gut bifidobacteria: Molecular strategies for their utilization. Food Microbiol. 2018;75:37–46. doi: 10.1016/j.fm.2017.09.001. 30056961

[73] Abdill RJ, Graham SP, Rubinetti V, Ahmadian M, Hicks P, Chetty A, McDonald D, Ferretti P, Gibbons E, Rossi M, et al. Integration of 168,000 samples reveals global patterns of the human gut microbiome. Cell. 2025;188:1100–1118. e17. doi: 10.1016/j.cell.2024.12.017. 39848248 PMC11848717

[74] Sindi AS, Geddes DT, Wlodek ME, Muhlhausler BS, Payne MS, Stinson LF. Can we modulate the breastfed infant gut microbiota through maternal diet? FEMS Microbiol Rev. 2021;45. doi: 10.1093/femsre/fuab011. 33571360

[75] Yeruva L, Mulakala BK, Rajasundaram D, Gonzalez S, Cabrera-Rubio R, Martinez-Costa C, Martínez-Costa C, Collado MC. Human milk miRNAs associate to maternal dietary nutrients, milk microbiota, infant gut microbiota and growth. Clin Nutr. 2023;42:2528–2539. doi: 10.1016/j.clnu.2023.10.011. 37931372

[76] Kramer MS, Kakuma R. Optimal duration of exclusive breastfeeding. Cochrane Database Syst Rev. 2012; 2012:CD003517. doi: 10.1002/14651858.CD003517.pub2. 11869667

[77] Sharp JA, Modepalli V, Enjapoori AK, Bisana S, Abud HE, Lefevre C, Nicholas KR. Bioactive functions of milk proteins: a comparative genomics approach. J Mammary Gland Biol Neoplasia. 2014;19:289–302. doi: 10.1007/s10911-015-9331-6. 26115887

