# Supplementary figures and images for: The triad of maternal gut-breast milk-infant gut microbial transmission in early life as a critical pathway for microbial inheritance
Source: Gut Microbes. 2025 Nov 16;17(1):2574928. doi: 10.1080/19490976.2025.2574928 (PMC12629333; doi:10.1080/19490976.2025.2574928)

Figure S1

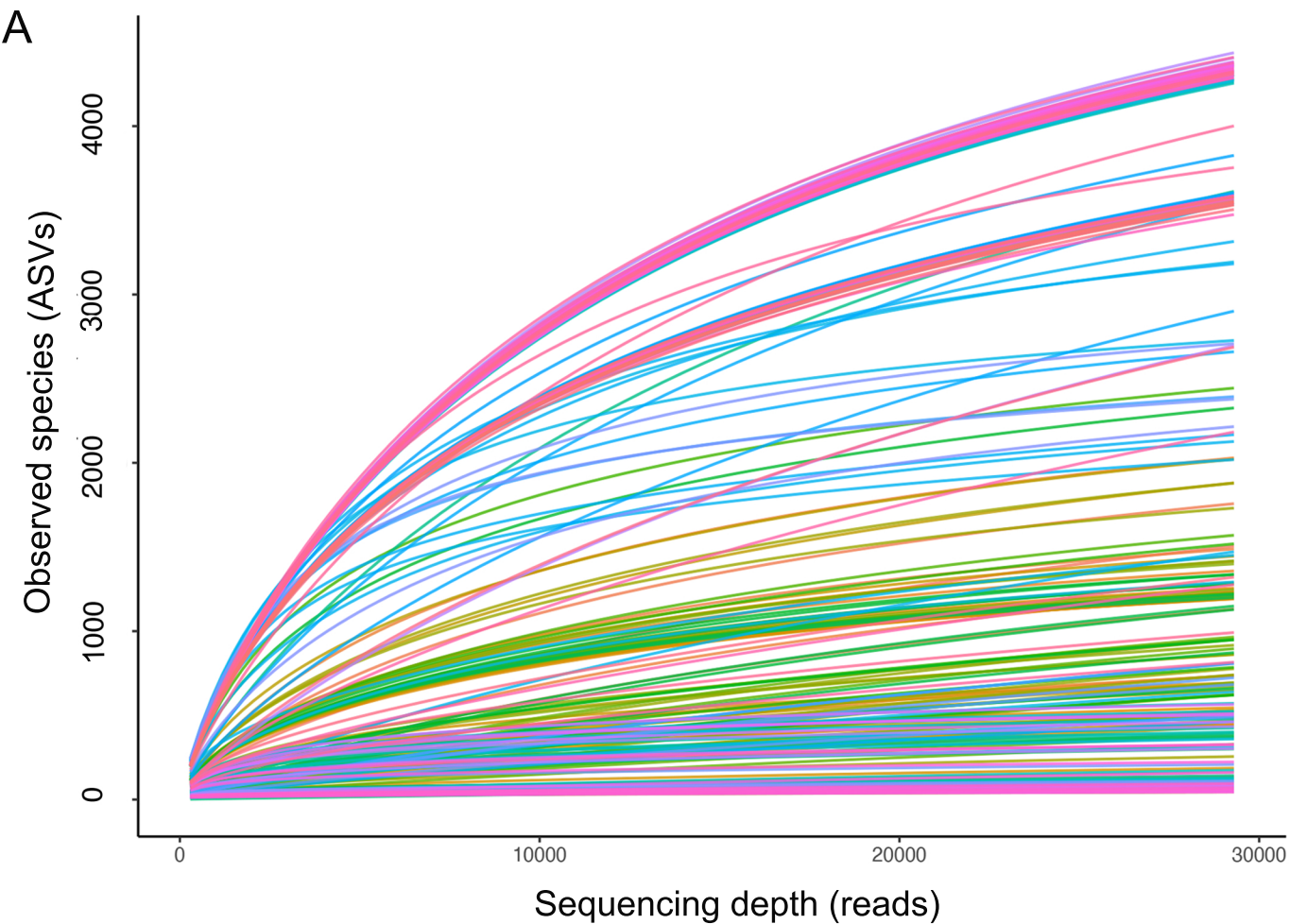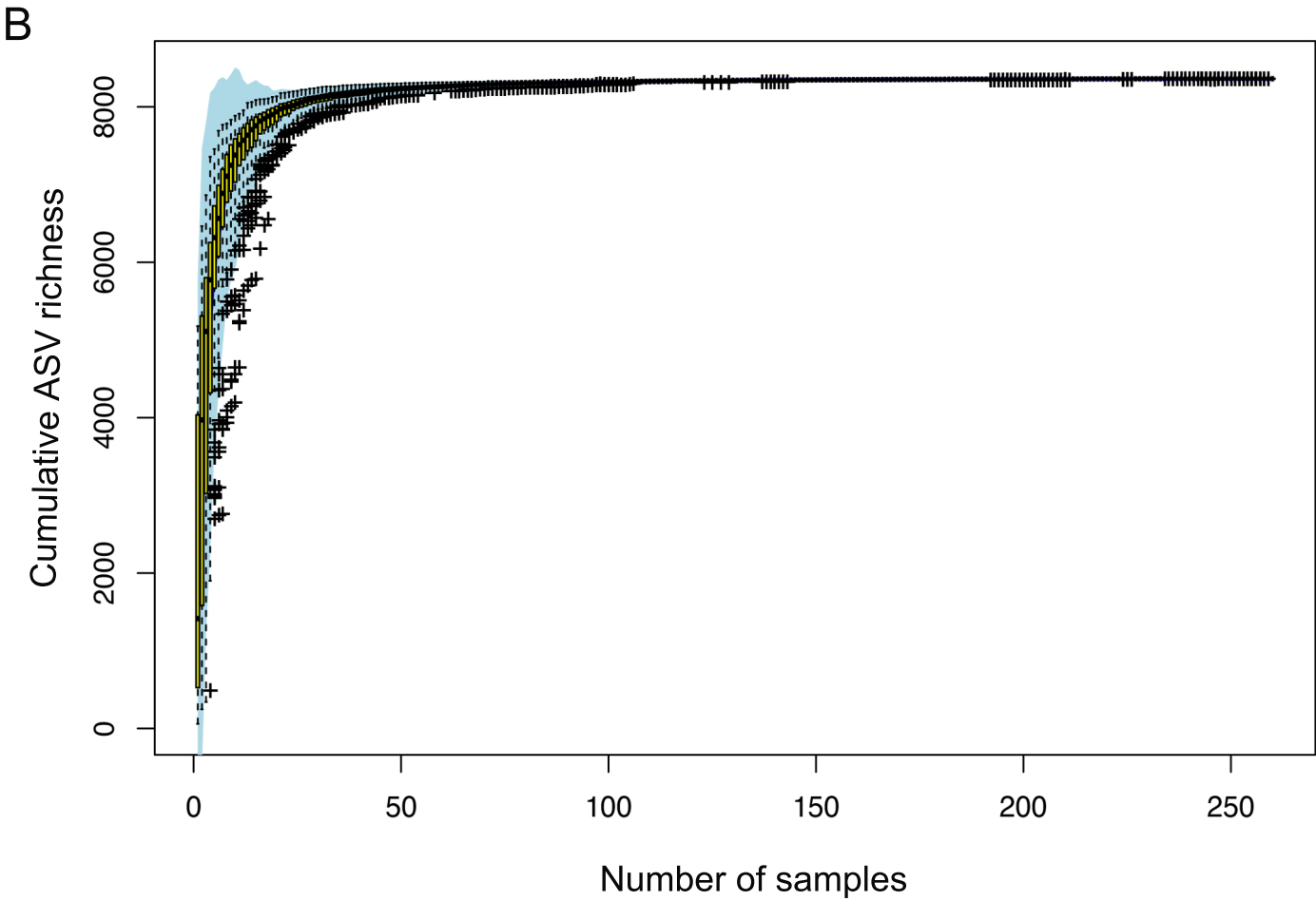

Supplement: Supplementary material — Supplementary Information. [file KGMI_A_2574928_SM2637.pdf]

Figure S4

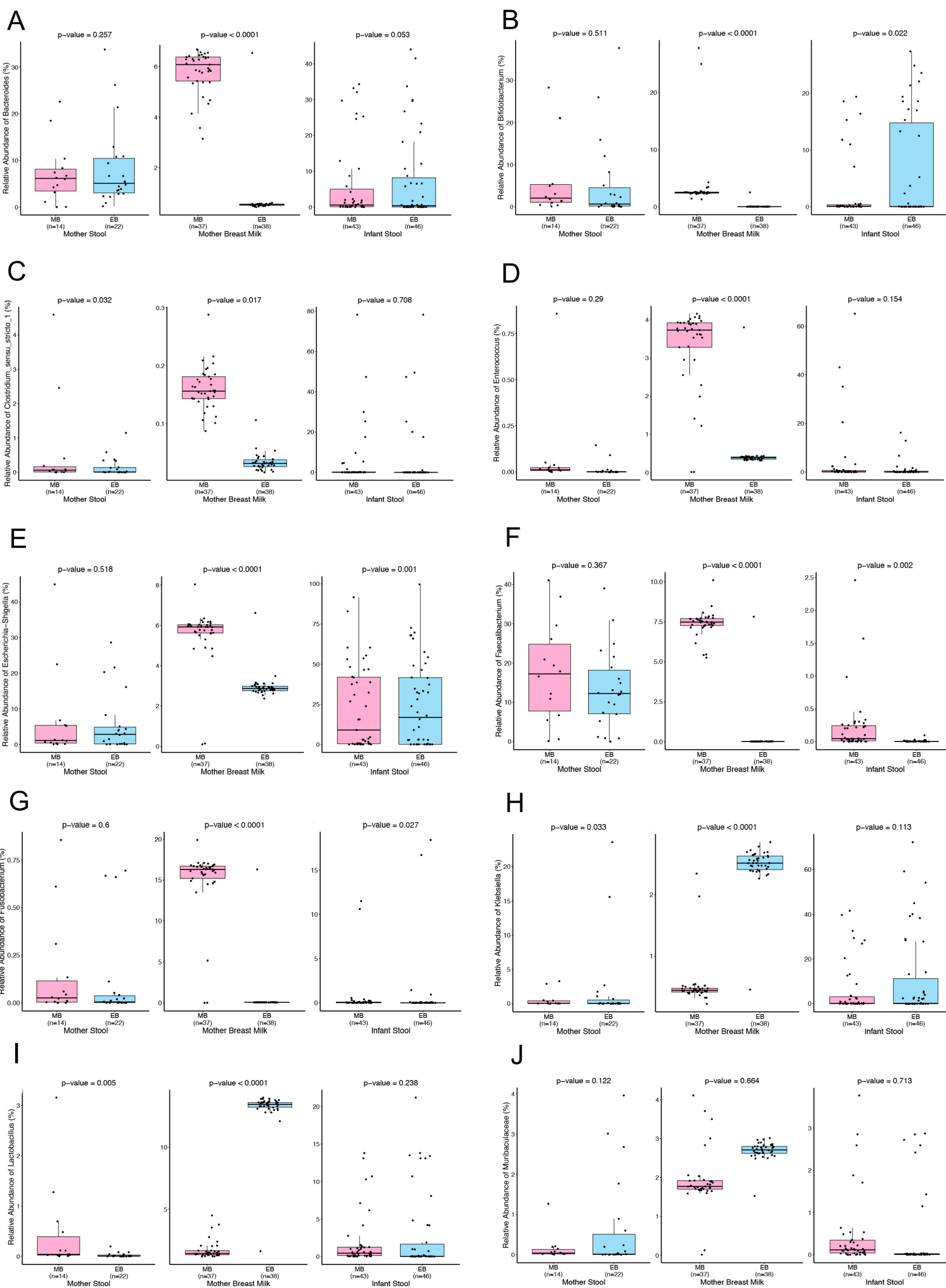

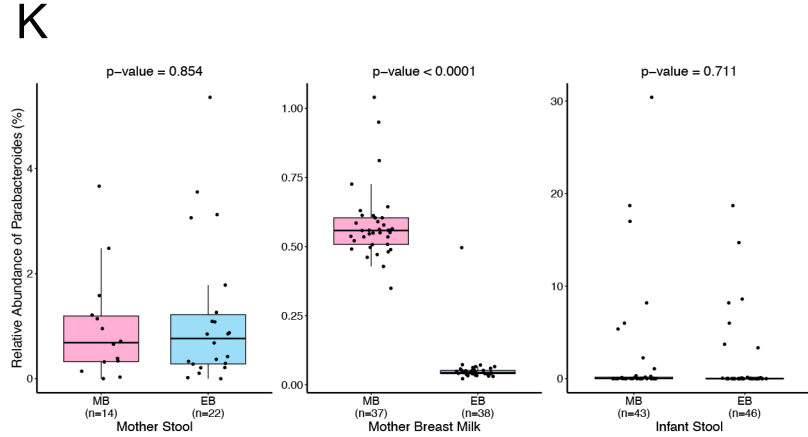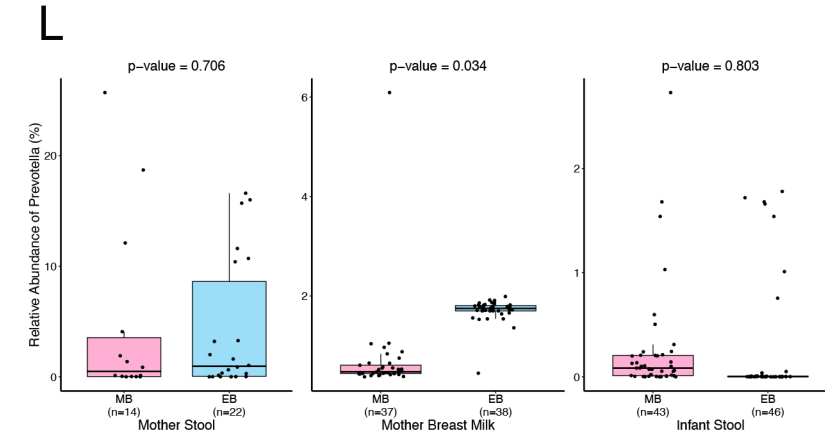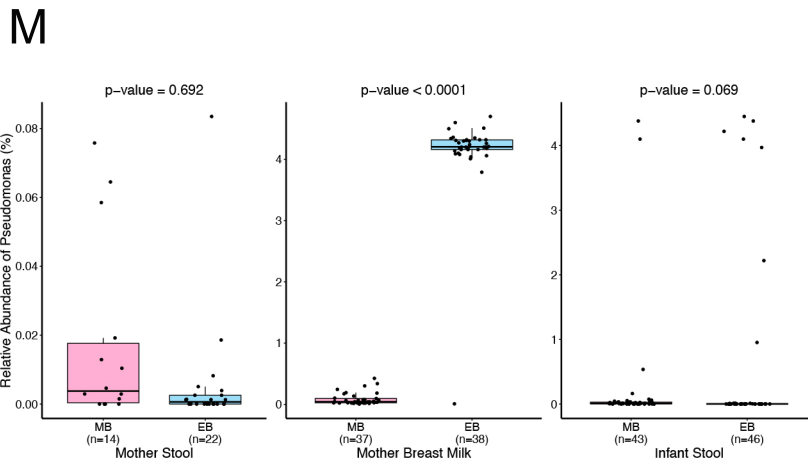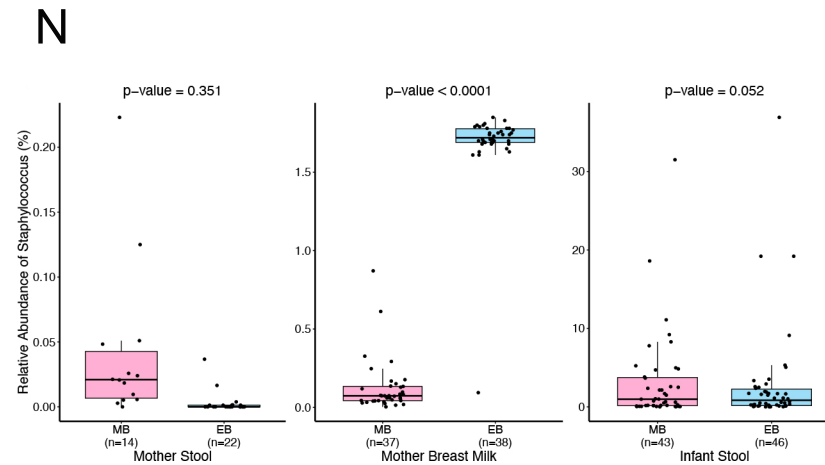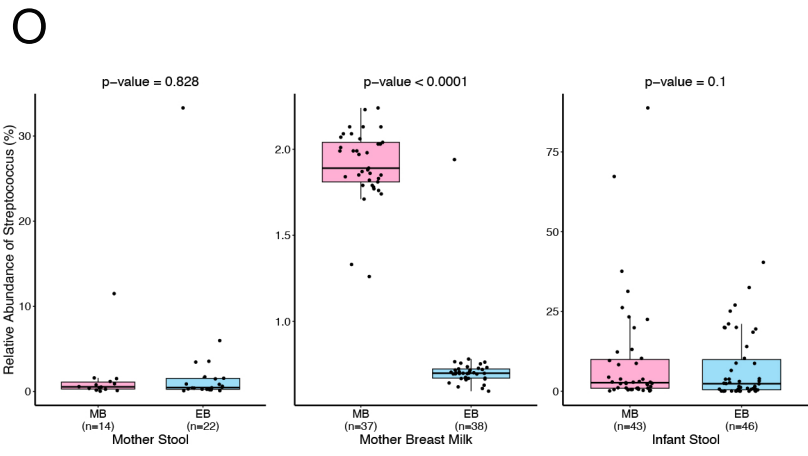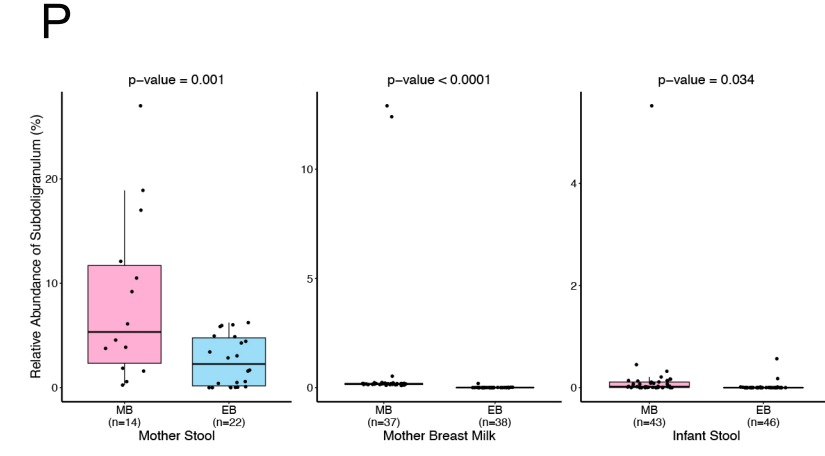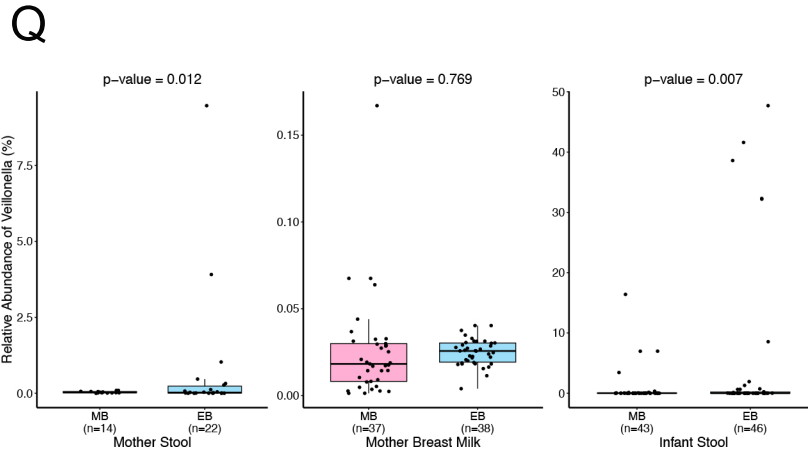

Supplement: Supplementary material — Supplementary Information. [file KGMI_A_2574928_SM2636.pdf]

Figure S2

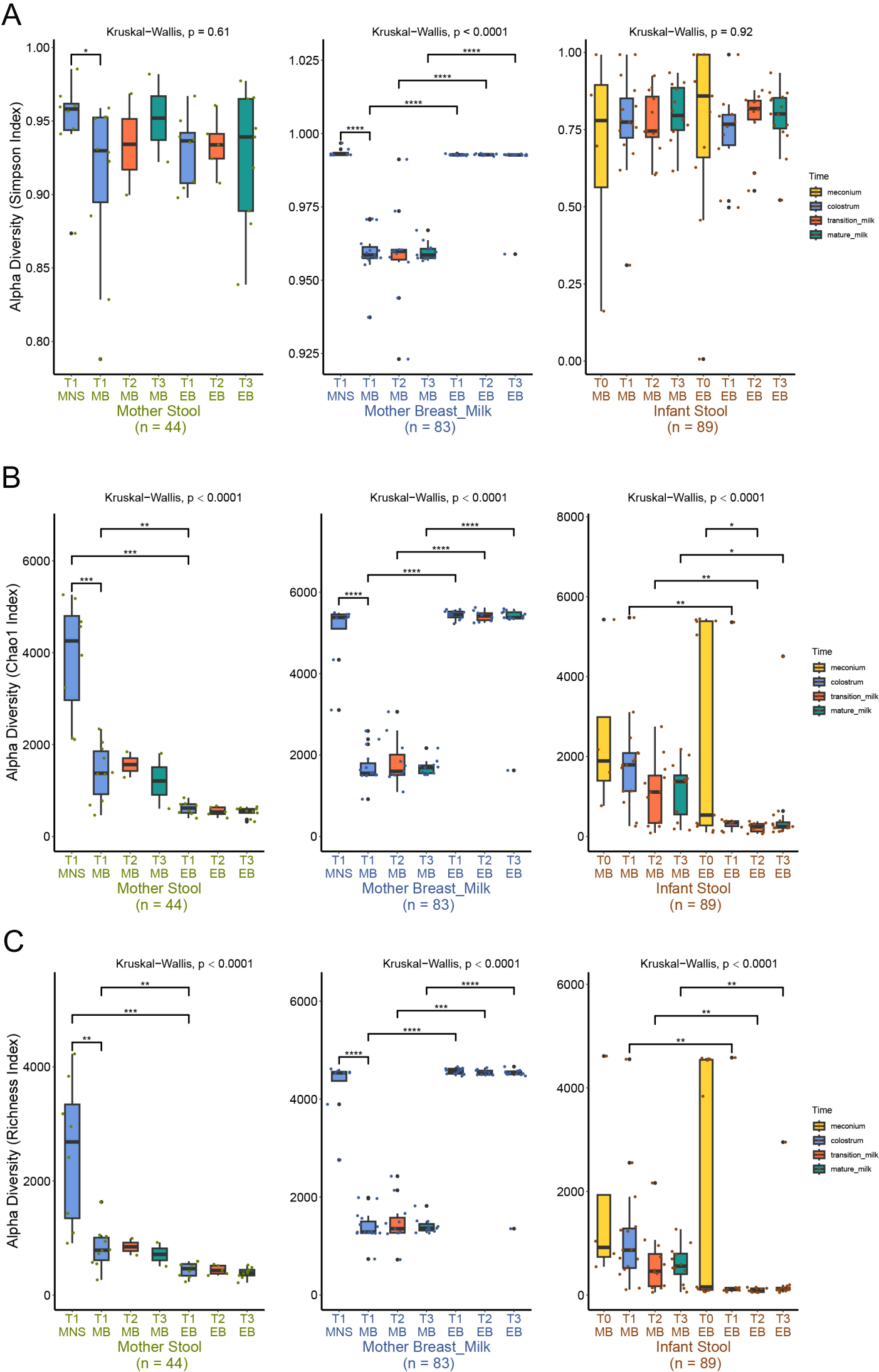

Supplement: Supplementary material — Supplementary Information. [file KGMI_A_2574928_SM2635.pdf]

Figure S3

A

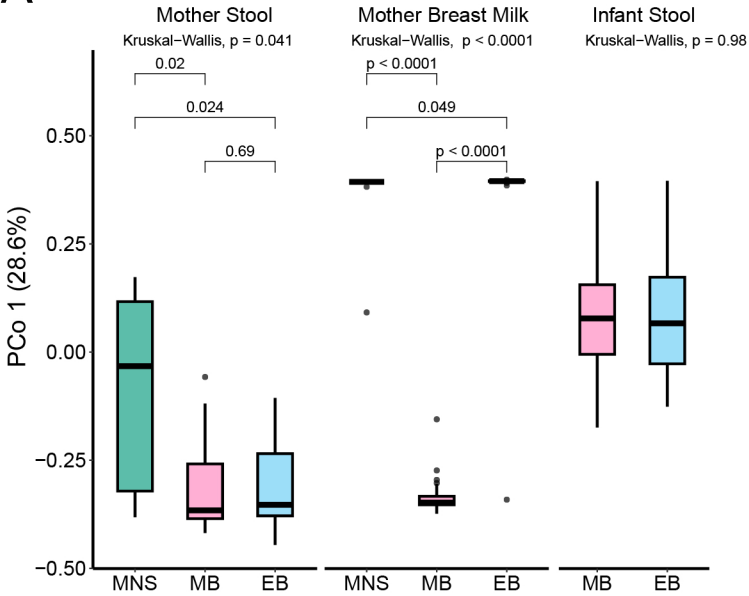

B

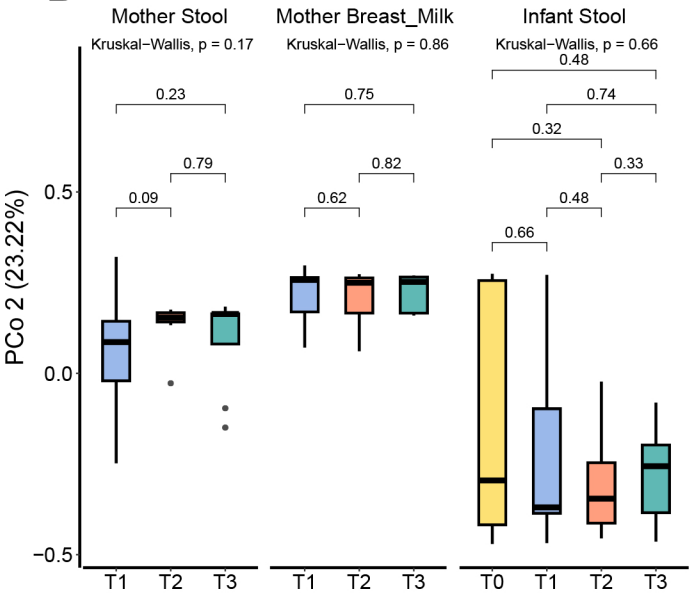

C

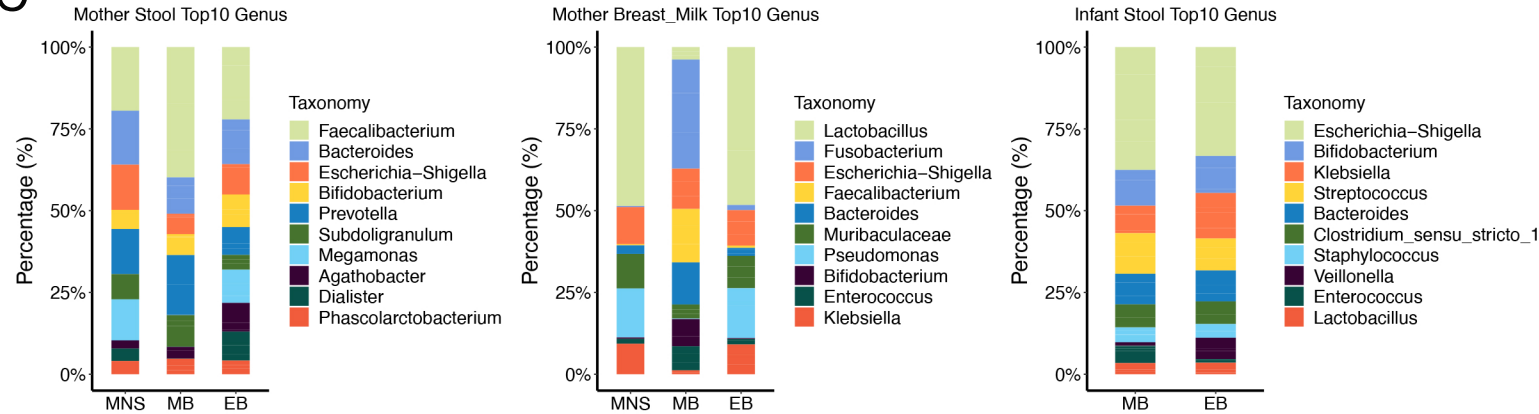

D

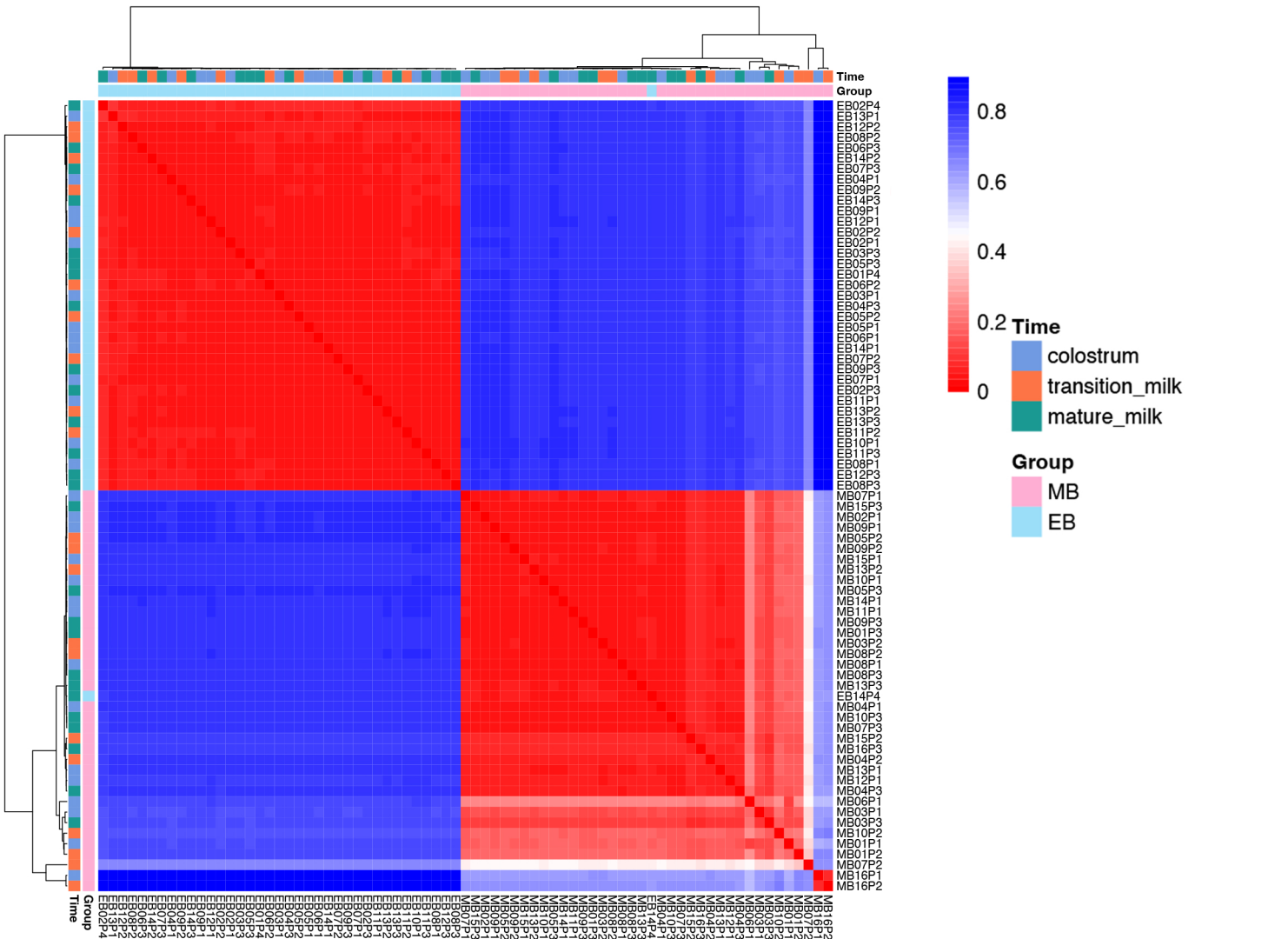

Supplement: Supplementary material — Supplementary Information. [file KGMI_A_2574928_SM2633.pdf]

Figure S6

A

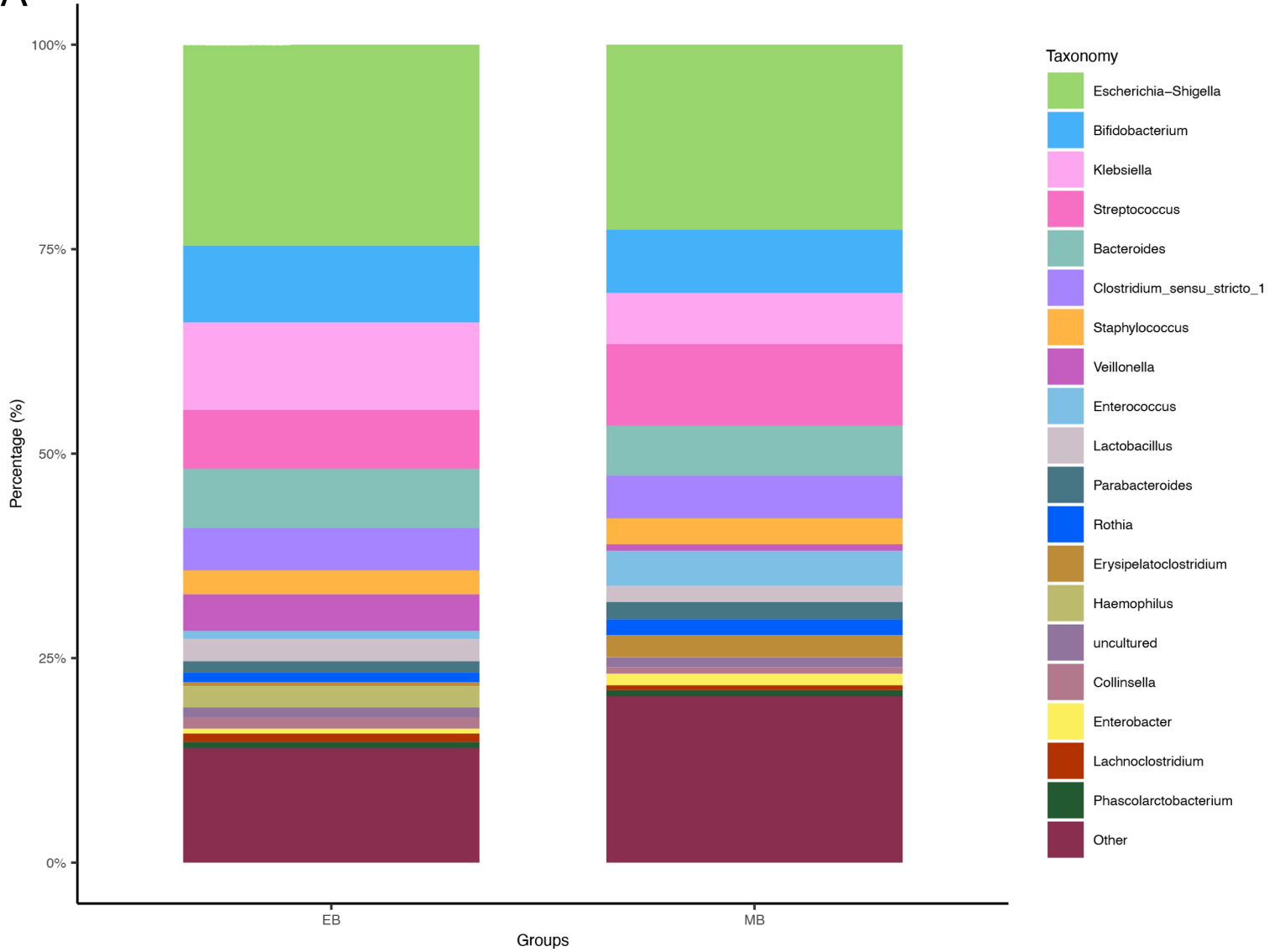

B

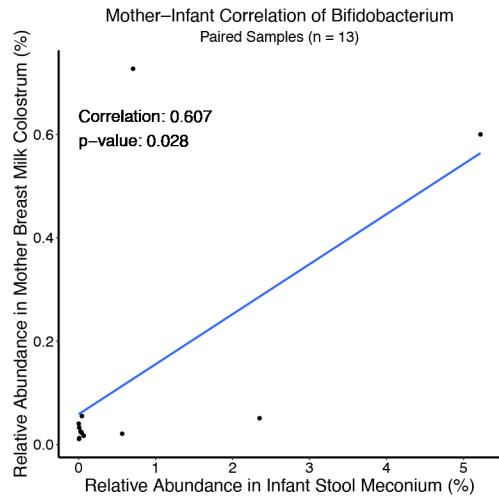

C

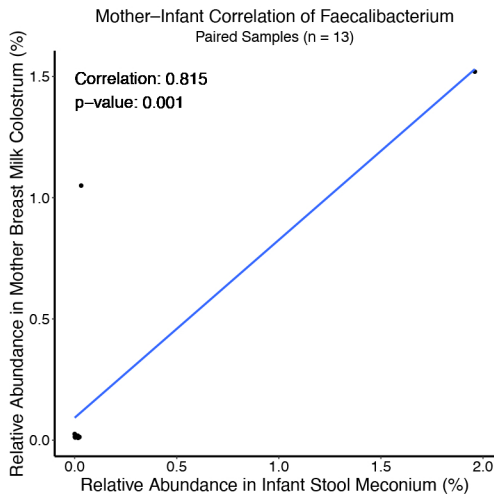

D

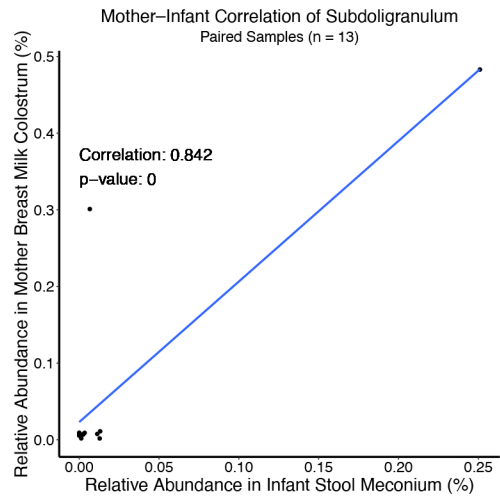

Supplement: Supplementary material — Supplementary Information. [file KGMI_A_2574928_SM2632.pdf]

## Figure S5

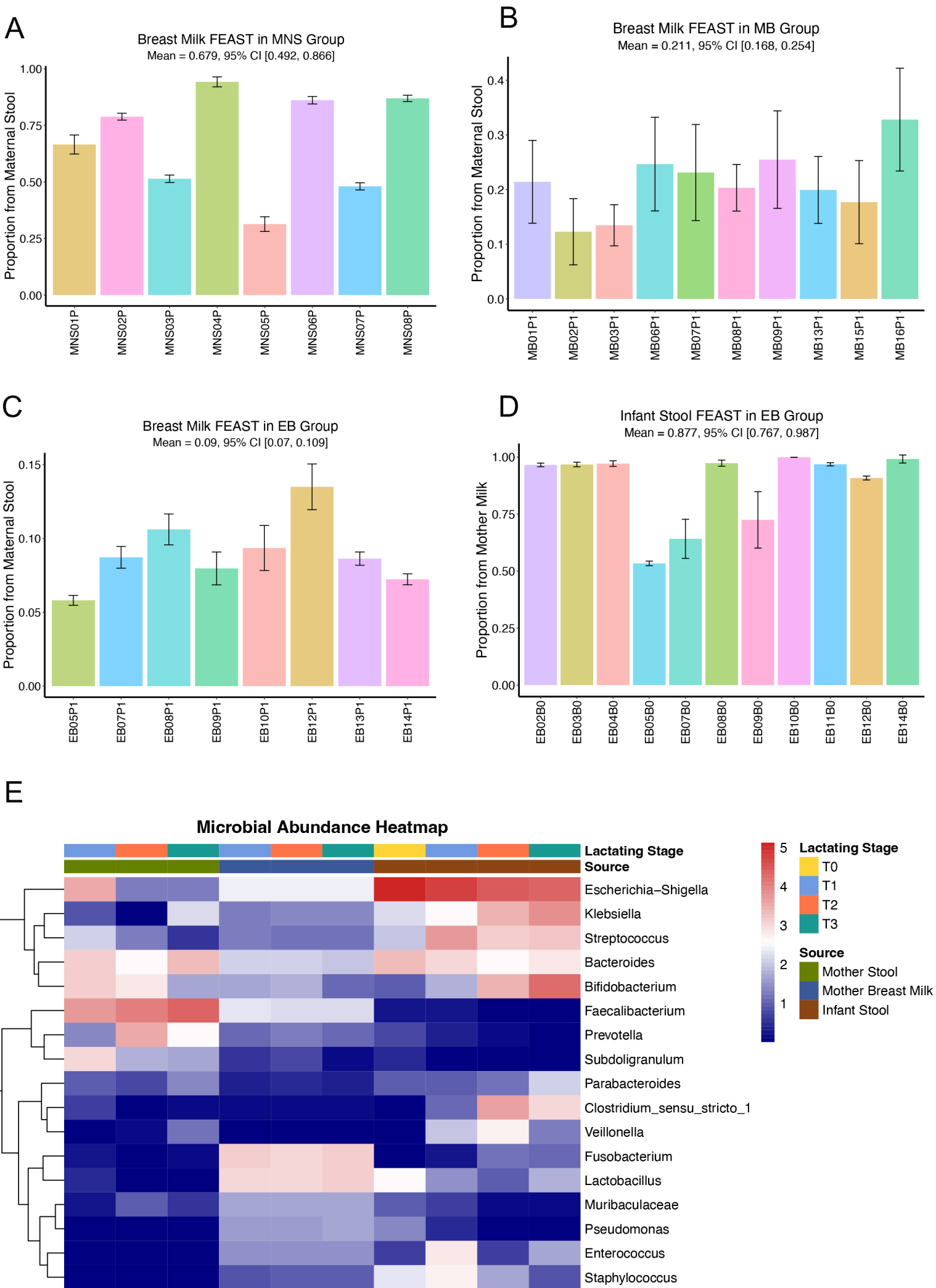

Supplement: Supplementary material — Supplementary Information. [file KGMI_A_2574928_SM2627.pdf]
