# Supplementary material for: The triad of maternal gut-breast milk-infant gut microbial transmission in early life as a critical pathway for microbial inheritance
Source: Gut Microbes. 2025 Nov 16;17(1):2574928. doi: 10.1080/19490976.2025.2574928 (PMC12629333; doi:10.1080/19490976.2025.2574928)
Supplement: Supplementary material — Table S1. The demographics of the participants. [file KGMI_A_2574928_SM2631.docx]

**Supplementary Material**

**Table S1. The demographics of the participants.**

| **Groups** | **Mother number** | **Mother Age**  **(years)** | **Parity** | **Gestational**  **Age (weeks＋days)** | **Delivery**  **Mode** | **Use of antibiotics during delivery** | **Maternal dietary during postpartum** | **Use of probiotics** | **Infant Gender** | **Infant** **Weight**  **(kg)** |
| --- | --- | --- | --- | --- | --- | --- | --- | --- | --- | --- |
| **1.1 Mother-newborn together (According to Feeding Method)**  **EB Group** | | | | | | | | | | |
| EB01 | 1 | 35 | 3 | 39+1 | Vaginal | No | LSLFHP Diet | No | Female | 3.55 |
| EB02 | 2 | 29 | 2 | 39+2 | Vaginal | No | LSLFHP Diet | No | Male | 3.50 |
| EB03 | 3 | 38 | 2 | 39 | C-section | No | Usual Diet | No | Male | 3.00 |
| EB04 | 4 | 26 | 1 | 40+5 | Vaginal | No | Usual Diet | No | Male | 3.55 |
| EB05 | 5 | 28 | 2 | 40 | Vaginal | No | LSLFHP Diet | No | Male | 3.59 |
| EB06 | 6 | 39 | 5 | 38+4 | C-section | No | LSLFHP Diet | No | Female | 3.34 |
| EB07 | 7 | 33 | 2 | 41 | Vaginal | No | LSLFHP Diet | No | Male | 3.55 |
| EB08 | 8 | 32 | 2 | 39+1 | C-section | No | Usual Diet | No | Female | 3.75 |
| EB09 | 9 | 33 | 2 | 41 | Vaginal | No | LSLFHP Diet | No | Male | 3.80 |
| EB10 | 10 | 33 | 1 | 40 | C-section | No | LSLFHP Diet | No | Male | 3.63 |
| EB11 | 11 | 37 | 2 | 39 | Vaginal | No | LSLFHP Diet | No | Male | 3.08 |
| EB12 | 12 | 28 | 3 | 38 | Vaginal | No | LSLFHP Diet | No | Female | 3.05 |
| EB13 | 13 | 35 | 2 | 39+5 | C-section | Yes | Usual Diet | No | Female | 3.40 |
| EB14 | 14 | 31 | 1 | 39+2 | Vaginal | Yes | LSLFHP Diet | No | Male | 3.73 |
| **MB Group** | | | | | | | | | | |
| MB01 | 15 | 31 | 2 | 40+1 | Vaginal | No | LSLFHP Diet | No | Male | 3.4 |
| MB02 | 16 | 31 | 2 | 37+6 | C-section | No | LSLFHP Diet | No | Male | 2.4 |
| MB03 | 17 | 35 | 3 | 39 | C-section | No | Usual Diet | No | Female | 3.85 |
| MB04 | 18 | 33 | 1 | 37+3 | Vaginal | No | LSLFHP Diet | No | Male | 3.0 |
| MB05 | 19 | 27 | 1 | 40 | Vaginal | No | Usual Diet | No | Male | 3.3 |
| MB06 | 20 | 33 | 3 | 38+4 | Vaginal | No | Usual Diet | No | Female | 3.0 |
| MB07 | 21 | 31 | 2 | 39+2 | Vaginal | No | LSLFHP Diet | No | Male | 3.0 |
| MB08 | 22 | 32 | 1 | 39+3 | Vaginal | No | Usual Diet | No | Female | 2.6 |
| MB09 | 23 | 32 | 2 | 40+5 | Vaginal | Yes | LSLFHP Diet | No | Male | 3.8 |
| MB10 | 24 | 33 | 1 | 39+6 | Vaginal | Yes | LSLFHP Diet | No | Female | 3.15 |
| MB11 | 25 | 39 | 2 | 39+1 | C-section | Yes | LSLFHP Diet | No | Male | 2.70 |
| MB12 | 26 | 30 | 1 | 39 | C-section | No | LSLFHP Diet | No | Female | 3.40 |
| MB13 | 27 | 31 | 2 | 39+2 | Vaginal | No | LSLFHP Diet | No | Male | 4.0 |
| MB14 | 28 | 35 | 2 | 38+6 | Vaginal | No | LSLFHP Diet | No | Male | 3.18 |
| MB15 | 29 | 35 | 3 | 40+2 | Vaginal | No | Usual Diet | No | Male | 3.10 |
| MB16 | 30 | 23 | 1 | 41 | Vaginal | No | Usual Diet | No | Female | 3.43 |
| **1.2 Mother-Newborn Separation Group** | | | | | | | | | | |
| MNS01 | 31 | 36 | 1 | 38 | Vaginal | No | LSLFHP Diet | No | Male | 2.53 |
| MNS02 | 32 | 31 | 1 | 39+3 | Vaginal | No | Usual Diet | No | Female | 2.64 |
| MNS03 | 33 | 26 | 1 | 39+5 | Vaginal | Yes | LSLFHP Diet | No | Female | 3.10 |
| MNS04 | 34 | 35 | 2 | 37+2 | C-section | No | LSLFHP Diet | No | Male | 2.37 |
| MNS05 | 35 | 29 | 1 | 39+2 | Vaginal | Yes | Usual Diet | Yes | Female | 3.65 |
| MNS06 | 36 | 32 | 1 | 39+3 | Vaginal | No | LSLFHP Diet | No | Male | 3.03 |
| MNS07 | 37 | 32 | 1 | 37+6 | Vaginal | No | LSLFHP Diet | No | Female | 3.10 |
| MNS08 | 38 | 36 | 1 | 38 | C-section | Yes | LSLFHP Diet | No | Female | 2.30 |

Note：LSLFHP Diet：Low-Salt, Low-Fat, High-Protein Diet.
