# Supplementary material for: The triad of maternal gut-breast milk-infant gut microbial transmission in early life as a critical pathway for microbial inheritance
Source: Gut Microbes. 2025 Nov 16;17(1):2574928. doi: 10.1080/19490976.2025.2574928 (PMC12629333; doi:10.1080/19490976.2025.2574928)
Supplement: Supplementary material — Table S7. Confounding factor analysis. [file KGMI_A_2574928_SM2628.docx]

**Supplementary Material**

**Table S7. Confounding Factor Analysis.**

| **7.1 Multivariate Regression Analysis of the Mother Stool-Mother Milk Ratio（R²=0.172, Adj R² = 0.001, Model p=0.441）** | | | |
| --- | --- | --- | --- |
| **Variables** | **Estimate (SE)** | **t value** | **p value** |
| Maternal dietary during postpartum (Usual Diet) | 0.042 (0.045) | 0.931 | 0.360 |
| Use of intrapartum antibiotics during delivery (Yes) | 0.082 (0.048) | 1.719 | 0.096 |
| Mother Age | -0.000 (0.006) | -0.052 | 0.959 |
| Delivery Mode (Vaginal) | 0.077 (0.054) | 1.434 | 0.162 |
| Infant Weight | -0.036 (0.050) | -0.726 | 0.474 |
| Infant Gender (Male) | -0.007 (0.042) | -0.163 | 0.871 |
| **7.2 Multivariate Regression Analysis of the Mother Milk-Infant Stool Ratio（R²=0.324, Adj R²=-0.256, Model p=0.752）** | | | |
| **Variables** | **Estimate (SE)** | **t value** | **p value** |
| Maternal dietary during postpartum (Usual Diet) | 0.202 (0.206) | 0.980 | 0.360 |
| Use of intrapartum antibiotics during delivery (Yes) | -0.003 (0.026) | -0.101 | 0.922 |
| Mother Age | -0.150 (0.214) | -0.701 | 0.506 |
| Delivery (Vaginal) | 0.049 (0.272) | 0.180 | 0.862 |
| Infant Weight | -0.045 (0.185) | -0.242 | 0.816 |
| Infant Gender (Male) | 0.127 (0.158) | 0.805 | 0.447 |
