# Supplementary material for: The triad of maternal gut-breast milk-infant gut microbial transmission in early life as a critical pathway for microbial inheritance
Source: Gut Microbes. 2025 Nov 16;17(1):2574928. doi: 10.1080/19490976.2025.2574928 (PMC12629333; doi:10.1080/19490976.2025.2574928)
Supplement: Supplementary material — Table S6. Comparison of 17 genera (relative abundance > 1%) in the mother stool, breast milk, and infant stool between the EB and MB groups. [file KGMI_A_2574928_SM2626.docx]

**Supplementary Material**

**Table S6.** **Comparison of 17 genera (relative abundance >1%) in the Mother Stool, Breast Milk, and Infant Stool between the EB and MB group.**

| **Genera** | **log_2_FC** | **log_2_CPM** | **P-value** | **FDR** | **Level** | **Source** |
| --- | --- | --- | --- | --- | --- | --- |
| *Muribaculaceae* | -0.092 | 15.292 | 0.6639 | 0.9985 | NotSig | Mother Breast Milk |
| *Muribaculaceae* | 1.189 | 14.34 | 0.1215 | 0.9935 | NotSig | Mother Stool |
| *Muribaculaceae* | -0.16 | 13.099 | 0.7128 | 0.9996 | NotSig | Infant Stool |
| *Bacteroides* | -3.405 | 15.565 | 0.0000 | 0.0000 | Depleted | Mother Breast Milk |
| *Bacteroides* | 0.671 | 16.717 | 0.2574 | 0.9935 | NotSig | Mother Stool |
| *Bacteroides* | 1.472 | 17.249 | 0.0533 | 0.9996 | NotSig | Infant Stool |
| *Prevotella* | 0.62 | 14.868 | 0.0338 | 0.2466 | NotSig | Mother Breast Milk |
| *Prevotella* | -0.329 | 16.023 | 0.7060 | 0.9935 | NotSig | Mother Stool |
| *Prevotella* | -0.134 | 13.035 | 0.8032 | 0.9996 | NotSig | Infant Stool |
| *Lactobacillus* | 2.269 | 16.431 | 0.0000 | 0.0000 | Enriched | Mother Breast Milk |
| *Lactobacillus* | -1.927 | 14.197 | 0.0048 | 0.3549 | Depleted | Mother Stool |
| *Lactobacillus* | 0.859 | 15.169 | 0.2377 | 0.9996 | NotSig | Infant Stool |
| *Parabacteroides* | -2.297 | 14.395 | 0.0000 | 0.0000 | Depleted | Mother Breast Milk |
| *Parabacteroides* | 0.111 | 14.775 | 0.8536 | 0.9935 | NotSig | Mother Stool |
| *Parabacteroides* | 0.346 | 14.864 | 0.7111 | 0.9996 | NotSig | Infant Stool |
| *Streptococcus* | -1.783 | 14.912 | 0.0000 | 0.0000 | Depleted | Mother Breast Milk |
| *Streptococcus* | 0.144 | 15.064 | 0.8276 | 0.9935 | NotSig | Mother Stool |
| *Streptococcus* | 0.952 | 17.359 | 0.1001 | 0.9996 | NotSig | Infant Stool |
| *Pseudomonas* | 3.936 | 15.232 | 0.0000 | 0.0000 | Enriched | Mother Breast Milk |
| *Pseudomonas* | -0.15 | 14.097 | 0.6917 | 0.9935 | NotSig | Mother Stool |
| *Pseudomonas* | 0.928 | 13.01 | 0.0695 | 0.9996 | NotSig | Infant Stool |
| *Staphylococcus* | 2.435 | 14.728 | 0.0000 | 0.0000 | Enriched | Mother Breast Milk |
| *Staphylococcus* | -0.442 | 14.103 | 0.3509 | 0.9935 | NotSig | Mother Stool |
| *Staphylococcus* | 1.202 | 15.961 | 0.0521 | 0.9996 | NotSig | Infant Stool |
| *Veillonella* | -0.107 | 14.203 | 0.7687 | 0.9985 | NotSig | Mother Breast Milk |
| *Veillonella* | 2.565 | 14.45 | 0.0123 | 0.8175 | Enriched | Mother Stool |
| *Veillonella* | 3.03 | 15.975 | 0.0074 | 0.6615 | Enriched | Infant Stool |
| *Clostridium_*  *sensu_stricto_1* | -1.095 | 14.252 | 0.0174 | 0.1487 | Depleted | Mother Breast Milk |
| *Clostridium_*  *sensu_stricto_1* | -1.56 | 14.302 | 0.0322 | 0.9935 | Depleted | Mother Stool |
| *Clostridium_*  *sensu_stricto_1* | 0.428 | 17.48 | 0.7081 | 0.9996 | NotSig | Infant Stool |
| *Fusobacterium* | -5.105 | 16.455 | 0.0000 | 0.0000 | Depleted | Mother Breast Milk |
| *Fusobacterium* | -0.386 | 14.18 | 0.5997 | 0.9935 | NotSig | Mother Stool |
| *Fusobacterium* | 2.246 | 14.267 | 0.0271 | 0.9996 | Enriched | Infant Stool |
| *Enterococcus* | -3.047 | 15.134 | 0.0000 | 0.0000 | Depleted | Mother Breast Milk |
| *Enterococcus* | -0.64 | 14.118 | 0.2899 | 0.9935 | NotSig | Mother Stool |
| *Enterococcus* | -1.275 | 15.648 | 0.1542 | 0.9996 | NotSig | Infant Stool |
| *Bifidobacterium* | -4.902 | 15.156 | 0.0000 | 0.0000 | Depleted | Mother Breast Milk |
| *Bifidobacterium* | -0.498 | 16.115 | 0.5113 | 0.9935 | NotSig | Mother Stool |
| *Bifidobacterium* | 1.906 | 17.229 | 0.0220 | 0.9996 | Enriched | Infant Stool |
| *Faecalibacterium* | -4.922 | 15.71 | 0.0000 | 0.0000 | Depleted | Mother Breast Milk |
| *Faecalibacterium* | -0.492 | 17.469 | 0.3666 | 0.9935 | NotSig | Mother Stool |
| *Faecalibacterium* | -1.485 | 13.025 | 0.0021 | 0.2941 | Depleted | Infant Stool |
| *Subdoligranulum* | -3.722 | 14.464 | 0.0000 | 0.0000 | Depleted | Mother Breast Milk |
| *Subdoligranulum* | -1.943 | 16.015 | 0.0005 | 0.0901 | Depleted | Mother Stool |
| *Subdoligranulum* | -1.038 | 13.03 | 0.0345 | 0.9996 | Depleted | Infant Stool |
| *Klebsiella* | 1.458 | 14.999 | 0.0000 | 0.0000 | Enriched | Mother Breast Milk |
| *Klebsiella* | 2.508 | 15.232 | 0.0326 | 0.9935 | Enriched | Mother Stool |
| *Klebsiella* | 1.356 | 17.802 | 0.1132 | 0.9996 | NotSig | Infant Stool |
| *Escherichia-Shigella* | -1.439 | 15.824 | 0.0000 | 0.0000 | Depleted | Mother Breast Milk |
| *Escherichia-Shigella* | -0.529 | 16.476 | 0.5182 | 0.9935 | NotSig | Mother Stool |
| *Escherichia-Shigella* | 2.084 | 18.846 | 0.0010 | 0.2403 | Enriched | Infant Stool |

Note: Enriched: Enriched in EB; Depleted: Enriched in MB; NotSig: No significance between EB and MB.
